# Research Progress of Photothermal Superhydrophobic Surfaces for Anti-Icing/Deicing

**DOI:** 10.3390/molecules30091865

**Published:** 2025-04-22

**Authors:** Hui Gao, Tianjun Yin, Jieyin Ma, Yuqin Zhou, Ke Li, Jiayi Bao

**Affiliations:** 1 School of Aeronautic Science and Engineering, Beihang University, Beijing 100191, China; h.gao@buaa.edu.cn (H.G.);; 2 College of Aeronautical Engineering, Civil Aviation University of China, Tianjin 300300, China

**Keywords:** photothermal, superhydrophobic surfaces, anti-icing/deicing, advanced coatings, applications

## Abstract

Photothermal superhydrophobic surfaces with micro/nano-structured morphologies have emerged as promising candidates for anti-icing and deicing applications due to their exceptional water repellency and efficient solar-to-thermal conversion. These surfaces synergistically integrate the passive icephobicity of superhydrophobic coatings with the active heating capability of photothermal materials, offering energy-efficient and environmentally friendly solutions for sectors such as aviation, wind energy, and transportation. Hence, they have received widespread attention in recent years. This review provides a comprehensive overview of recent advances in photothermal superhydrophobic coatings, focusing on their anti-icing/deicing mechanisms, surface wettability, and photothermal conversion performance for anti-icing/deicing applications. Special emphasis is placed on material categories, including metals and their compounds, carbon-based materials, and polymers, analyzing their structural features and application effectiveness. Furthermore, the application of anti-icing/deicing in various fields is described. Finally, perspectives on future development are presented, including pursuing fluorine-free, cost-effective, and multifunctional coatings to meet the growing demand for innovative, sustainable anti-icing/deicing technologies.

## 1. Introduction

Ice can negatively impact various areas, including wind turbines, aircraft, transportation, etc. [1,2,3,4,5,6,7]. For example, aircraft icing, caused by supercooled water droplets freezing on surfaces, distorts aerodynamics, increases drag, reduces lift, and impairs control, with glaze ice posing more severe risks than rime ice [1,2,3]. Similarly, ice on wind turbine blades alters aerodynamic profiles, increases structural loads, and reduces efficiency [6,7], while for vehicles, icing adds weight, disrupts stability, and impairs sensors, heightening safety risks. Consequently, effective anti-icing and deicing strategies are critical for ensuring safety, preserving equipment performance, and extending operational lifespan, particularly in cold-climate regions.

Traditional anti-icing/deicing methods, such as mechanical removal, chemical spraying, and hot gas/electric heating, can be used for different application scenarios but still face the problems of aerodynamic shape damage, environmental pollution, or high energy consumption [8,9,10]. To address these challenges, various effective and eco-friendly anti-icing and deicing technologies, such as icephobic coatings, slippery liquid-infused porous surfaces (SLIPS), acoustic wave systems, and solar-absorbing materials, have attracted increasing attention. Among them, superhydrophobic surfaces and photothermal material systems have been extensively proposed and developed over the past few decades [3,11,12,13,14,15,16]. Studies indicate that superhydrophobic surfaces can effectively suppress ice formation and reduce ice adhesion due to their low wettability and surface energy, facilitating easy ice removal from these surfaces with low energy costs [11,12,13,14]. However, under prolonged exposure to high humidity and extremely low temperatures, superhydrophobic surfaces tend to transition from the Cassie–Baxter state to the Wenzel state, resulting in inevitable icing, increased ice adhesion, and diminished anti-icing/deicing effectiveness [15,16]. As for photothermal coatings, composed of carbon nanotubes, Fe_3_O_4_, and Ag nanoparticles, they can absorb sunlight and convert it into heat, helping to prevent icing or melting the ice that forms on them. When superhydrophobic coatings are integrated together with photothermal materials, photothermal superhydrophobic coatings are formed; these could effectively combine the advantages of superhydrophobicity and photothermal effects [17,18]. These coatings not only exhibit low surface energy and reduced ice adhesion but also efficiently convert sunlight into thermal energy to increase surface temperature [17,18,19,20]. This dual action mechanism significantly enhances their anti-icing and deicing performance. Furthermore, photothermal superhydrophobic coatings offer an environmentally friendly and energy-efficient alternative to traditional methods, with advantages such as durability and chemical resistance, which unveil promising future applications [17,18,19,20].

**Figure 1:**
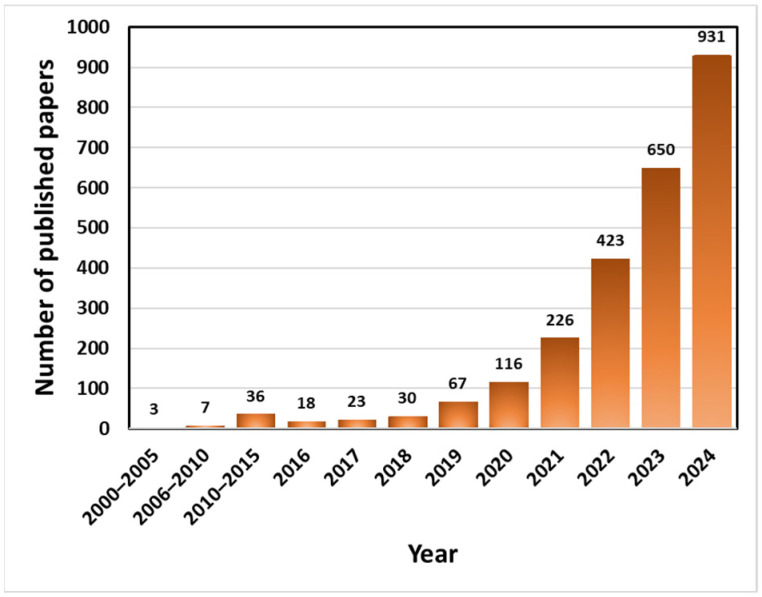
Number of recently published academic papers on photothermal superhydrophobic materials for anti-icing/deicing listed in Web of Science.

Due to their outstanding potential for anti-icing and deicing applications, there has been a growing research interest in photothermal superhydrophobic surfaces in recent years. As shown in Figure 1, the number of academic papers published on the topic has increased quickly year by year, and especially in 2024 alone, 931 works addressing photothermal superhydrophobic surfaces were published. The number of review papers associated with this topic is also increasing; however, they have primarily concentrated on the applications of either photothermal or superhydrophobic surfaces in anti-icing and deicing [17,21]. Review articles on photothermal superhydrophobic surfaces are rare, and most of them mainly explore using photothermal superhydrophobic surfaces in areas such as seawater desalination, oil–water separation, and thermal management [22,23,24]. Hence, systematic and in-depth reviews on photothermal superhydrophobic materials for anti-icing and deicing application are still needed to bridge the gap.

Herein, this review begins by summarizing the fundamental icing theories, the principles of icephobicity, and the anti-icing/deicing mechanisms associated with superhydrophobic and photothermal coatings. Subsequently, substrate materials commonly utilized for photothermal superhydrophobic systems are summarized, highlighting the relationships between surface structure and photothermal anti-icing/deicing performance across various surface types. Finally, this paper outlines the current challenges, prospective applications, and directions for advancing photothermal superhydrophobic coatings in anti-icing/deicing technologies.

## 2. Mechanism of Ice Formation and Photothermal Superhydrophobic Anti-Icing/Deicing

### 2.1. Ice Formation: Nucleation and Structural Diversity

**Figure 2:**
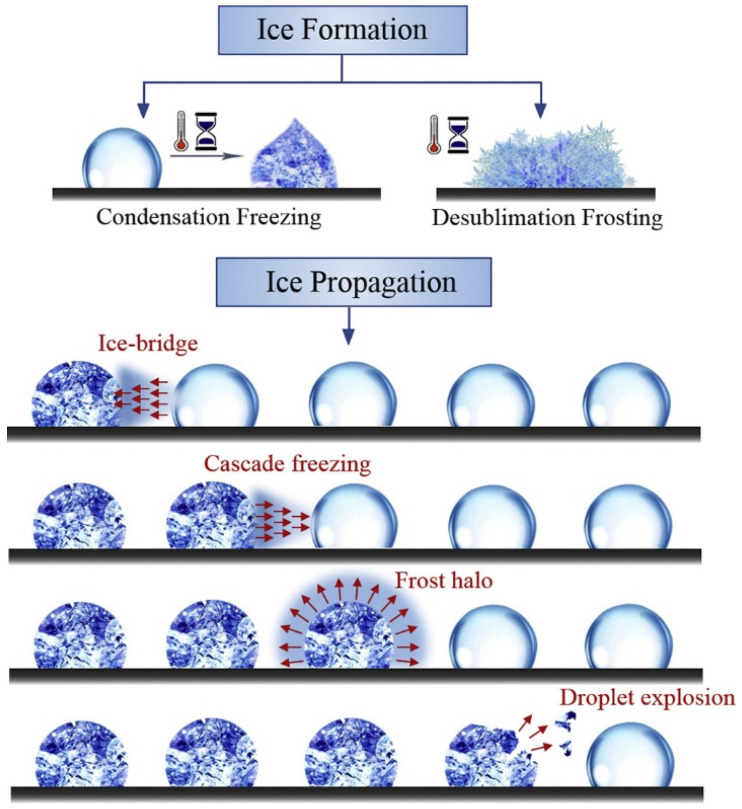
Ice formation and propagation mechanism [31].

Ice accretion, the buildup of ice and snow on exposed surfaces, occurs as ice nuclei, primarily atmospheric dust particles (~70%), facilitating the freezing of supercooled water droplets [25,26]. The activation of ice nuclei, which varies with temperature, initiates ice crystal growth and precipitation in mixed-phase clouds, where ice crystals grow at the expense of supercooled droplets due to saturation vapor pressure differences, eventually becoming heavy enough to descend while undergoing accretion, melting, coalescence, or evaporation [25,26,27]. As temperature decreases, more ice nuclei activate. Then, it initiates a four-stage freezing process between ice nuclei and water droplets, promoting ice crystal development through the Wegener–Bergeron–Findeisen process [26,27], as shown in Figure 2. Ice crystals contribute to various atmospheric phenomena, including clouds, precipitation, and halos [27,28,29,30,31]. Hexagonal ice needles form in the upper atmosphere at −5 °C, while ice crystal growth morphology depends on humidity: high humidity favors plate-like structures, dry conditions promote columnar shapes, and intermediate levels lead to dendritic snowflakes [27,28,29,30]. Generally, natural freezing phenomena can be categorized as glaze, wet snow, rime, and hoarfrost [32,33,34,35,36,37,38,39,40,41,42,43].

In harsh environments, ice accumulation presents significant hazards. For instance, ice on roadways in cold temperatures can lead to car accidents, while ice on aircraft can alter aerodynamics, increasing flight risks and threatening human life and property [44]. Additionally, freezing electrical wires, coupled with the weight of rime and wind, can cause power infrastructure to collapse, disrupting the power supply and posing further safety risks [45,46,47]. Consequently, developing effective and efficient anti-icing/deicing strategies in industrial production, transportation, power transmission, and other fields is crucial.

### 2.2. Anti-Icing/Deicing Mechanism of Superhydrophobic Materials

**Figure 3:**
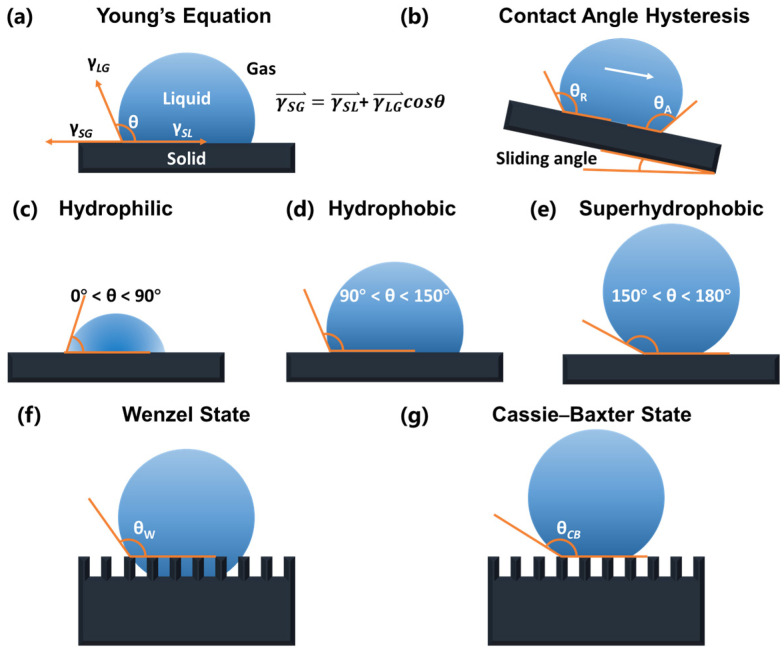
(**a**) Young’s equation; (**b**) contact angle hysteresis and sliding angle; (**c**) hydrophilic, (**d**) hydrophobic, and (**e**) superhydrophobic surface state; (**f**) Wenzel state; (**g**) Cassie–Baxter state.

Superhydrophobic surfaces, characterized by high contact angles, low sliding angles, and low surface energy, significantly influence icing behavior by reducing ice adhesion and enhancing anti-icing and deicing performance, making them a key research focus [48,49]. These surfaces are commonly observed in nature; examples include lotus leaves and butterfly wings [50,51]. In 1756, Johann Gottlob Leidenfrost observed that when a liquid comes into contact with a surface significantly hotter than its boiling point (>200 °C), a vapor layer forms between the liquid and the surface, preventing direct contact, which is known as the Leidenfrost effect [52,53,54]. Under these conditions, the liquid droplet exhibits a contact angle approaching 180°, with negligible contact angle hysteresis [54,55]. In the 19th century, Thomas Young formulated the fundamental theorem of contact angles, known as Young’s equation, which determines the shape of liquid droplets on solid surfaces and applies to equilibrium states on homogeneous surfaces without specific interactions between the solid and liquid phases [56,57,58]. Young’s equation describes the relationship between the interfacial tensions <solid–gas ($\gamma_{SG}$), solid–liquid ($\gamma_{SL}$), and liquid–gas ($\gamma_{LG}$)> and the contact angle (θ) [56,57]. This equation (shown in Figure 3a), also referred to as the wetting equation, is expressed as follows:

(1) $$\gamma_{SG}=\gamma_{SL}+\gamma_{LG}cos\theta$$

The contact angle (CA, *θ*) ranges from 0° to 180°, indicating the surface’s wettability characteristics [54,55,56,57,58]. As illustrated in Figure 3c–e, surfaces with contact angles (θ) below 90° are hydrophilic, those with contact angles above 90° are hydrophobic, and when θ exceeds 150°, the surface is considered superhydrophobic, exhibiting extreme water repellency [58,59,60,61,62]. Moreover, as shown in Figure 3b, contact angle hysteresis refers to the difference between a liquid’s advancing and receding contact angles on a solid surface [63,64]. This phenomenon arises due to surface roughness, chemical heterogeneity, or contaminants, which cause the contact line to become “pinned”, impeding its movement [63,64]. The sliding angle (rolling or roll-off angle) is the minimum tilt angle required for a droplet to begin moving or rolling off a surface [65].

In addition to Young’s equation, the Wenzel and Cassie–Baxter models are commonly used to characterize superhydrophobic surfaces [66,67,68,69]. In 1936, Robert N. Wenzel found that surface roughness increases the solid–liquid contact area beneath a droplet, raising interfacial energy and reducing wettability by enhancing water repellency [67,68,69]. The Wenzel equation indicates that, in the Wenzel state (Figure 3f), the apparent contact angle ($\theta_{w}$) is amplified relative to the intrinsic contact angle (θ) under specific conditions [70]. The modified Wenzel equation is expressed as follows:

(2) $$cos\theta_{w}=rcos\theta_{\gamma }$$

where $\theta_{w}$ is the apparent contact angle in Wenzel’s theory, and *r* (*r* > 1) is the surface roughness ratio. Here, r is obtained as follows:

(3) $$r=\frac{\sigma_{r}}{\sigma_{0}}=\frac{cos\theta_{w}}{cos\theta_{\gamma }}$$

where $\sigma_{r}$ is the ratio of the actual contact area of the solid and the geometric surface, and $\sigma_{0}$ is the projected area of the contact area of the solid–liquid surface. In 1944, Cassie and Baxter extended the concept of surface roughness affecting contact angles. They observed that on surfaces of porous mediums, the liquid cannot penetrate the grooves of the rough surface, leaving air voids [66,71,72]. Consequently, they formulated the Cassie–Baxter equation (shown in Figure 3g):

(4) $$cos\theta_{CB}=\sigma_{1}cos\theta_{1}-\sigma_{2}=\sigma_{1}\left(cos\theta_{1}+1\right)-1$$

where $\theta_{1}$ is the intrinsic contact angle on a smooth, homogeneous surface of the same material, compared to the apparent contact angle on a rough or heterogeneous surface, $\theta_{CB}$. Later, in 1948, Cassie improved the calculation method for two materials with different chemical properties on smooth and rough surfaces, creating Cassie’s law [73,74]:

(5) $$cos\theta_{C}=\sigma_{1}cos\theta_{1}+\sigma_{2}cos\theta_{1}$$

where $\theta_{1}$ and $\theta_{2}$ are the contact angles associated with the two distinct surface components, and $\sigma_{1}$ and $\sigma_{2}$ are the area fractions of these components, satisfying $\sigma_{1}+\sigma_{2}=1$.

Due to surface roughness, droplets on superhydrophobic surfaces often undergo heterogeneous nucleation during freezing, but the reduced solid–liquid contact area enhances anti-icing and deicing performance [12,17,58,75]. Superhydrophobic surfaces typically feature micro/nano-scale hierarchical structures that induce the Cassie–Baxter state, in which air pockets form a liquid–solid–gas tri-phase interface [73,76,77,78]. This minimizes direct contact between droplets and the solid substrate, reducing potential nucleation sites and increasing the nucleation-free energy barrier, thereby delaying ice formation [76,77,78,79]. In contrast, hydrophilic surfaces promote freezing due to close droplet–substrate contact and increased nucleation sites from surface defects. Furthermore, the air layer on superhydrophobic surfaces lowers thermal conductivity and increases thermal resistance, hindering the heat exchange between the droplet and the substrate. As a result, droplets cool more slowly under supercooled conditions, further inhibiting or delaying ice nucleation [12,58,77].

In practical anti-icing/deicing applications, dynamic wetting transitions between Cassie–Baxter and Wenzel states play a crucial role. While micro/nano-structured surfaces promote water repellency via the Cassie–Baxter state, environmental factors such as condensation and frost melting can cause water to infiltrate surface textures, inducing a transition to the Wenzel state with increased solid–liquid contact and ice adhesion [70,72]. This is especially problematic during freeze–thaw cycles, where refreezing water anchors ice within the roughness. As reported, such transitions are often irreversible and significantly reduce icephobicity [17]. Therefore, anti-icing/deicing surface designs must consider static wettability and wetting stability under dynamic conditions to ensure long-term performance.

### 2.3. Anti-Icing/Deicing Mechanism of Photothermal Conversion Materials

**Figure 4:**
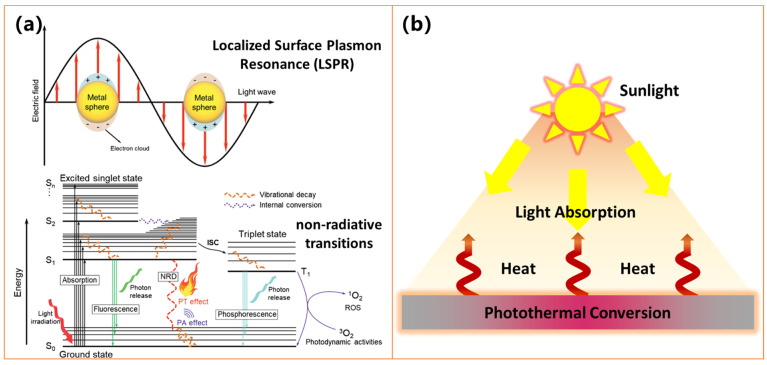
Schematic diagram of (**a**) photon-to-heat conversion, including LSPR and non-radiative transitions [86], and (**b**) macroscopic photothermal conversion process.

Photothermal surfaces exhibit superior applicability in anti-icing and deicing [24,80,81,82,83]. They utilize photothermal conversion materials to absorb solar energy and convert it into thermal energy, thereby heating the surface to prevent the formation of ice layers or melt the formed ice (Figure 4) [80,81,82,83]. The core of this technology lies in an efficient photothermal conversion process, i.e., the ability to transform light energy into thermal energy [80,81,82,83]. This process primarily involves the following mechanisms and processes:

Light Absorption [84,85]: Photothermal materials absorb incident solar radiation. The efficiency of this absorption depends on the material’s properties and the design of light-trapping structures, which can enhance solar energy absorption by altering the propagation direction of light and inducing frustrated total internal reflection.

Photon-to-Heat Conversion [85,86,87]: As shown in Figure 4a, electrons are excited to higher energy levels when materials absorb photon energy. These high-energy electrons transfer energy to the lattice through non-radiative transitions, enhancing lattice vibrations and increasing temperature, thereby converting light energy into thermal energy. In materials such as metal nanoparticles, incident light induces collective oscillations of free electrons, known as localized surface plasmon resonance (LSPR) [87]. This resonant effect enhances light absorption efficiency, and the oscillating electrons convert kinetic energy into thermal energy through damping, leading to a localized temperature increase. In certain semiconductor materials, excited electrons release energy in the form of heat through non-radiative relaxation processes, increasing material temperature after absorbing photons.

Heat Transfer [88,89]: The generated thermal energy is conducted through the material’s bulk and interfaces, leading to a temperature rise at the surface. This localized heating can effectively melt existing ice or prevent the nucleation and growth of ice crystals. When the temperature reaches or exceeds the melting point of ice, melting initiates. Alternatively, maintaining the temperature above the freezing point can prevent ice formation.

Based on this mechanism, an ideal photothermal anti-icing/deicing material should possess several key properties [83,90,91,92]:

High Solar Absorptance: The material should efficiently absorb solar radiation across a broad spectral range to maximize energy intake.

High Photothermal Conversion Efficiency: It should effectively convert the absorbed light into thermal energy, ensuring rapid heating.

Effective Thermal Radiation Management: The material should minimize heat loss through optimized thermal emissivity, maintaining the necessary temperature for deicing.

Durability and Environmental Adaptability: It should withstand environmental factors such as temperature fluctuations, moisture, and mechanical wear, ensuring long-term performance.

### 2.4. Anti-Icing/Deicing Mechanism of Photothermal Superhydrophobic Materials

**Figure 5:**
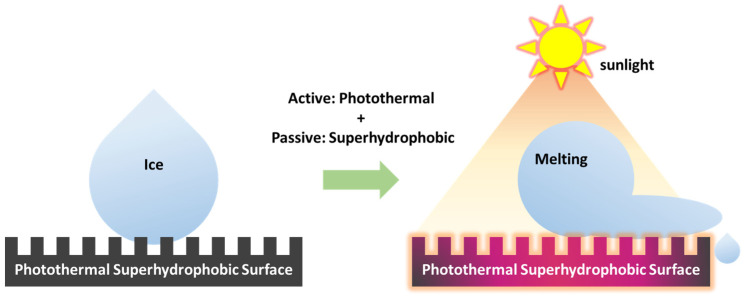
Schematic diagram of the anti-icing/deicing process of photothermal superhydrophobic surfaces.

As shown in Figure 5, by combining superhydrophobic surfaces with photothermal materials, the resulting composite surface synergistically integrates passive ice repellency and active photothermal heating, offering superior anti-icing and deicing performance and adaptability through the following integrated mechanisms [62,91,92,93,94,95,96,97,98]:

Active ice prevention: The photothermal effect actively raises the surface temperature above the freezing point, thereby hindering the nucleation and growth of ice crystals.

Passive ice prevention: The superhydrophobic Cassie–Baxter state minimizes liquid–solid contact, significantly reducing the probability of ice formation and accumulation.

Reduced ice adhesion and rapid ice removal: Even under harsh conditions where ice may form, the superhydrophobic structure’s inherently low ice adhesion strength, coupled with photothermal-induced interfacial melting, facilitates rapid ice detachment or melting.

In a nutshell, photothermal superhydrophobic surfaces exploit the synergistic interaction between low contact area, reduced ice adhesion characteristics (passive mechanisms), and efficient photothermal heating (active mechanisms), leading to remarkable improvements in overall anti-icing and deicing performance under severe environmental conditions.

## 3. Advances in Photothermal Superhydrophobic Surfaces for Anti-Icing/Deicing

A wide range of materials have been employed in the fabrication of photothermal superhydrophobic surfaces for anti-icing and deicing. To impart photothermal properties to superhydrophobic surfaces, various photothermal materials have been utilized. They could be broadly categorized into three types: metals and their compounds [19,22,24,81,99,100,101,102,103,104,105,106,107,108,109,110,111,112,113,114,115,116,117,118,119,120], carbon-based materials [24,99,121,122,123,124,125,126,127,128,129,130,131,132,133,134], and polymers [135,136,137,138,139,140,141,142]. For the sake of brevity and clarity, this section provides a detailed overview of photothermal superhydrophobic materials based on the above three categories.

### 3.1. Metal-Based and Metallic-Compound-Based Photothermal Superhydrophobic Materials

Metals and metallic compounds, characterized by exceptional light absorption, thermal stability, and mechanical strength, play a pivotal role in photothermal superhydrophobic coatings [19,22,24,81,99,100,101,102,103,104,105,106,107,108,109,110,111,112,113,114,115,116,117,118,119,120]. This section provides a comprehensive analysis of metals (e.g., copper, silver, gold, iron, and aluminum) and metallic compounds (e.g., CuO, CuS, TiN, and Fe_3_O_4_), highlighting their distinct properties in anti-icing/deicing [22,24,81,99,100,101,102,103,104,105,106,107,108,109,110,111,112,113,114,115,116,117,118,119,120].

#### 3.1.1. Metal-Based

Metals with plasmonic properties (e.g., copper, silver, gold) could efficiently absorb and convert solar energy into heat through the collective oscillation of free electrons, enhancing their photothermal anti-icing/deicing performance [81,100,104]. Their excellent thermal stability allows them to withstand high-temperature variations without structural degradation, making them ideal for fluctuating environments [105,106,107,108,109]. Additionally, metals like aluminum and copper provide mechanical strength and corrosion resistance, ensuring the durability of superhydrophobic coatings [110]. Laser surface treatment can enhance superhydrophobicity by creating hierarchical micro/nanostructures that improve light trapping [104,106]. Table 1 presents the details of recent studies on metal-based photothermal superhydrophobic surfaces. The specific research content will be elaborated in subsequent sections.

##### Copper

Copper efficiently converts solar energy into heat, particularly with surface modifications such as laser treatments or nanoparticle coatings, leveraging its strong plasmonic properties for enhanced light absorption [100,101,102,103]. Its high thermal conductivity enables rapid, uniform heating for effective ice and frost removal. With hydrophobic coatings, copper gains corrosion resistance, ensuring durability in harsh environments. Compared to other metals, copper’s affordability makes it a cost-effective choice for large-scale anti-icing and deicing applications [81,104]. Recent advancements in copper-based photothermal superhydrophobic materials have demonstrated significant potential in anti-icing/deicing applications [24,104]. A common feature among these studies is the integration of hierarchical micro-/nano-structures with tailored surface functionalization, resulting in exceptional superhydrophobicity (water contact angles exceeding 150°) and efficient photothermal conversion [81,103]. Furthermore, these materials exhibit robust stability under extreme environmental conditions, mechanical wear, and chemical exposure, underscoring their suitability for practical implementation.

**Figure 6:**
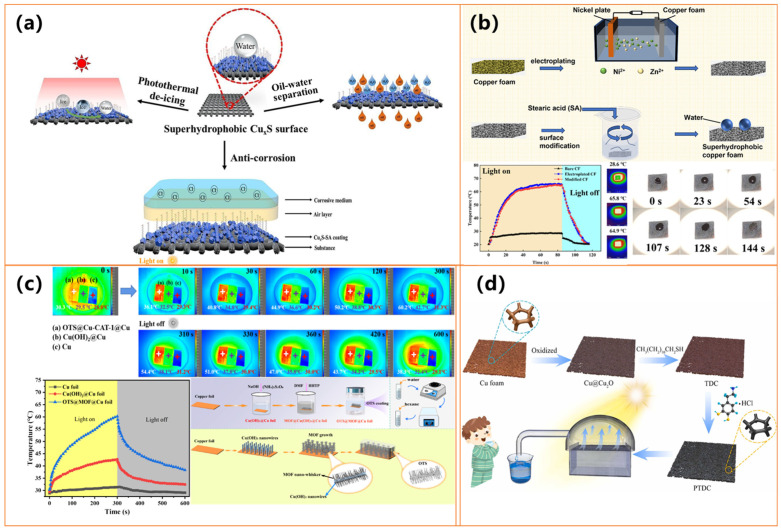
(**a**) Characterization of Cu@Cu_x_S-SA mesh [100]; (**b**) preparation and photothermal properties of superhydrophobic copper foam [101]; (**c**) preparation and photothermal properties of Cu-CAT-1@CM and the oil–water separation process [102]; (**d**) preparation process of PTDC membrane and its application in solar water purification [103].

Despite these overarching similarities, differences in anti-icing/deicing property complexity are evident. Wang et al. [100] employed a one-step initiated chemical vapor deposition (iCVD) approach to fabricate transparent superhydrophobic coatings with well-defined nanocone arrays, achieving high optical transmittance (~94.5%) and substantial anti-icing performance (icing delay of 540 s), as shown in Figure 6a. Conversely, Wu et al. [101] developed a cost-effective, two-step electroplating and chemical modification process to construct copper foam structures which exhibited pronounced photothermal heating (up to ~64.9 °C under solar irradiation) and superior oil adsorption efficiency, making it highly suitable for marine oil spill remediation, as shown in Figure 6b.

He et al. [102] introduced an environmentally benign, fluorine-free strategy to synthesize hierarchical Cu-based nanostructures, achieving exceptional anti-icing properties, including prolonged ice formation delay (870 s) and efficient photothermal deicing (surface temperature reaching ~60.2 °C within 300 s under sunlight exposure). As shown in Figure 6c, their study also incorporated molecular dynamics simulations to elucidate the underlying mechanisms governing ice adhesion reduction. Meanwhile, Dong et al. [103] fabricated a multifunctional copper-based photothermal membrane through an oxidation-assisted process followed by dual-functional surface modifications, as shown in Figure 6d. This resulted in high photothermal conversion efficiency (~92%), enhanced corrosion resistance, and outstanding water purification performance, including desalination and heavy metal removal. These studies collectively highlight the potential of photothermal superhydrophobic materials for environmental and energy-related applications. The variations in fabrication techniques, surface modifications, and functional attributes underscore the versatility and adaptability of these materials, paving the way for their widespread deployment in practical scenarios.

##### Silver

In addition to copper, silver is widely used in photothermal superhydrophobic materials due to its strong plasmonic response, which enhances solar energy absorption and photothermal conversion efficiency [105,106,107]. Its ability to reflect and absorb light further improves performance. Silver’s intrinsic antibacterial properties also benefit self-cleaning applications, making silver-based materials highly suitable for advanced multifunctional uses [105,106,107]. Recent advancements highlight their applicability in anti-icing/deicing, oil spill remediation, and protective coatings. These materials commonly integrate hierarchical micro- or nanostructures with functionalized surfaces, achieving exceptional water repellency (WCA > 150°), high photothermal conversion efficiency, and durability under extreme conditions [105,106,107].

**Figure 7:**
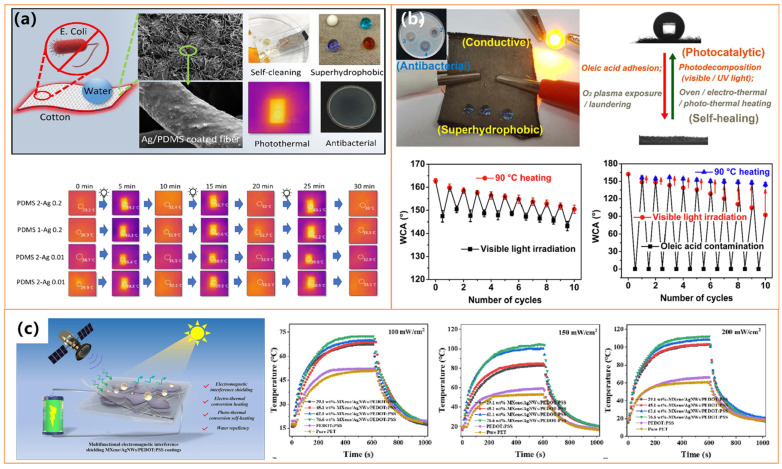
(**a**) Characterization of the multifunctional and photothermal properties of Ag/PDMS coatings [105]; (**b**) photothermal properties of superhydrophobic and conductive cotton fabric [106]; (**c**) electrothermal/photothermal mechanism and photothermal properties of MXene/AgNWs/PEDOT:PSS [107].

Pakdel et al. [105] and Li et al. [106] developed superhydrophobic textiles with Ag-based nanoparticles, leveraging photothermal effects for deicing and self-healing. Pakdel et al. [105] incorporated PDMS and Ag NPs, achieving rapid surface heating (46.5 °C) under near-infrared irradiation, effectively delaying ice formation, as shown in Figure 7a. Li et al. [106] enhanced photothermal performance by integrating Ag/CdS nanoparticles, significantly increasing electrothermal heating capacity (153.2 °C) under solar exposure, as shown in Figure 7b. This enabled the self-restoration of hydrophobicity and the degradation of organic contaminants, demonstrating a synergistic effect between photothermal activity and functional durability.

Beyond textiles, as shown in Figure 7c, Chang et al. [107] engineered ultrathin EMI shielding coatings using MXene, silver nanowires, and PEDOT: PSS, achieving enhanced hydrophobicity (WCA ~120.2°) alongside superior photothermal conversion. These coatings reached ~69.4 °C under one-sun illumination, highlighting their potential for passive deicing and thermal management applications. Their ability to combine high thermal response with electromagnetic shielding (~31.5 dB) and environmental stability makes them highly suitable for harsh and cold environments. All these studies underscore the versatility of photothermal superhydrophobic materials, demonstrating their effectiveness in energy-efficient deicing, self-cleaning, and environmental protection. These materials present promising opportunities for next-generation protective technologies and thermal management solutions by optimizing photothermal conversion properties with hydrophobicity.

##### Gold

**Figure 8:**
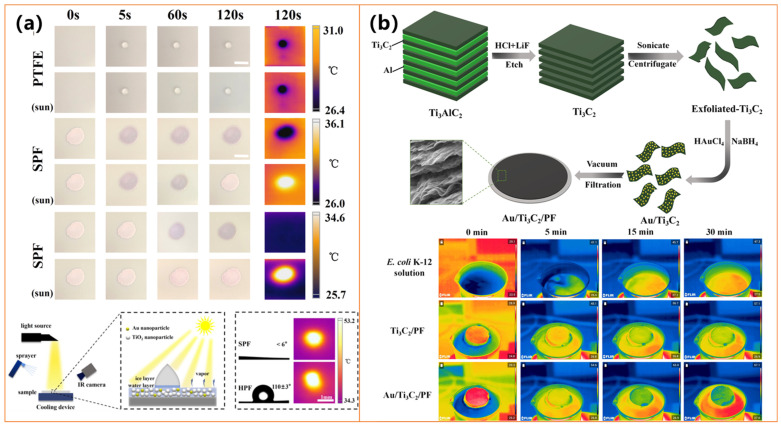
(**a**) Temperature distribution of Au/TiO_2_ plasmonic films under light illumination of 3 suns [109]; (**b**) fabrication and IR thermal images of 3.42% Au/Ti_3_C_2_/PF system [19].

Gold, as a precious metal, plays a crucial role in photothermal superhydrophobic coatings due to its ability to enhance light absorption, improve heat conversion efficiency, provide antibacterial properties, and enhance surface hydrophobicity and stability [108,109,110]. Its strong plasmonic resonance effect makes it highly effective in photothermal applications, enabling the development of multifunctional coatings for anti-icing, deicing, water purification, and defogging applications [108,109,110]. Wang et al. [108] developed a superhydrophobic photothermal composite coating by embedding MXene and AuNPs into a WPU matrix, achieving a water contact angle of 153° and reaching 130 °C under NIR light. This coating significantly extended the anti-icing time to 1053 s at −20 °C and achieved a deicing efficiency of 73.1%, making it highly effective in preventing ice accumulation and ensuring rapid ice removal. Similarly, Tao et al. [109] fabricated Au/TiO_2_ plasmonic films with both superhydrophilic and hydrophobic versions, where the hydrophobic film (HPF) efficiently prevented ice formation and enabled rapid deicing, increasing the surface temperature by over 25 °C under solar irradiation, as shown in Figure 8a. These coatings capitalize on the plasmonic resonance effect of gold to optimize light absorption and heat conversion, ensuring long-term stability in harsh conditions.

Expanding beyond anti-icing applications, Qu et al. [19] developed a multifunctional Au/Ti_3_C_2_ photothermal membrane for solar-driven water purification. This membrane exhibited excellent photothermal performance, achieving a high water evaporation rate of 2.66 kg/m^2^·h and an evaporation efficiency of 83.63% under solar illumination, as shown in Figure 8b. Additionally, it demonstrated strong antibacterial properties, completely inactivating *E. coli* and *Spingopyxis* sp. BM1-1 through the generation of singlet oxygen (^1^*O*_2_) and localized heating effects. While Wang et al. [108] and Tao et al. [109] focus on anti-icing and deicing, Qu et al. [110] highlight the versatility of gold-based photothermal materials in water purification. These studies underscore the synergy between superhydrophobicity and photothermal conversion, offering scalable and durable solutions for ice prevention, efficient deicing, and sustainable water purification in extreme environmental conditions.

##### Other Metals

**Figure 9:**
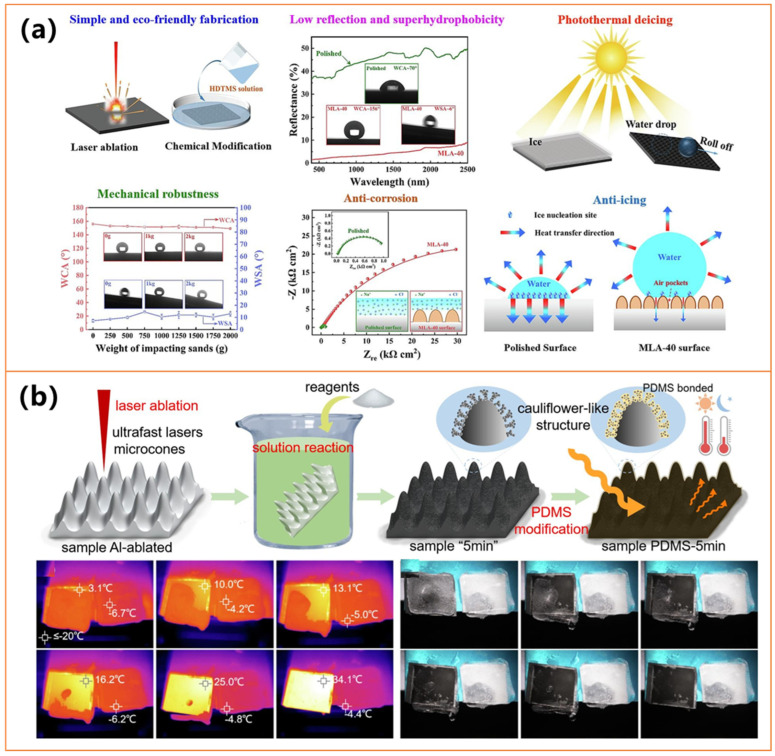
(**a**) Fabrication and anti-icing/deicing property of black superhydrophobic surface [110]; (**b**) fabrication and photothermal properties of durable cauliflower-like micro- or nanostructured superhydrophobic surface [112].

In addition to the previously discussed metals, recent studies on iron (Fe)- and aluminum (Al)-based coatings have demonstrated significant advancements in passive ice prevention and active ice removal by leveraging hierarchical micro/nanostructures, photothermal effects, and enhanced durability [93,110,111,112]. As shown in Figure 9, these coatings exhibit high water repellency (contact angles > 160°), effectively minimizing ice adhesion and delaying ice formation [93,110,111,112]. Simultaneously, their strong light absorption (>90%) enables rapid surface heating under sunlight, achieving temperature increases of 48–52 °C within minutes, facilitating efficient deicing. Moreover, reducing ice adhesion strength allows for easier ice detachment, enhancing long-term anti-icing performance [93,110,111,112].

Despite these shared characteristics, different fabrication approaches contribute to distinct functional advantages in anti-icing/deicing. Iron-based composite coatings (e.g., Zheng et al. [93]) utilize a dual-layer PDMS-Fe–candle soot structure, incorporating magnetic responsiveness and self-healing properties, making them suitable for curved surfaces and harsh environmental conditions. Laser-structured steel surfaces (e.g., Li et al., as shown in Figure 9a [110]) achieve enhanced corrosion resistance and reduced reflectance, leading to superior photothermal efficiency and mechanical robustness. Meanwhile, aluminum-based coatings (e.g., Li et al. [111] and Chen et al., as shown in Figure 9b [112]) demonstrate exceptional light absorption (up to 97.3%), resulting in rapid ice melting even under low solar intensities and prolonged outdoor stability. Additionally, hierarchical microcone or cactus-like structures on aluminum substrates contribute to enhanced self-cleaning and long-term durability, making them highly suitable for real-world applications in extreme weather conditions. All these advancements underscore the effectiveness of photothermal superhydrophobic materials in simultaneously preventing ice formation and facilitating efficient deicing. Future research should focus on optimizing these coatings’ scalability, environmental sustainability, and long-term performance under dynamic weather conditions. Further exploration of multi-functional materials, including self-repairing and adaptive surfaces, could provide more sustainable and durable solutions for critical applications such as power transmission lines, aerospace components, and transportation infrastructure.

#### 3.1.2. Metallic-Compound-Based

**Figure 10:**
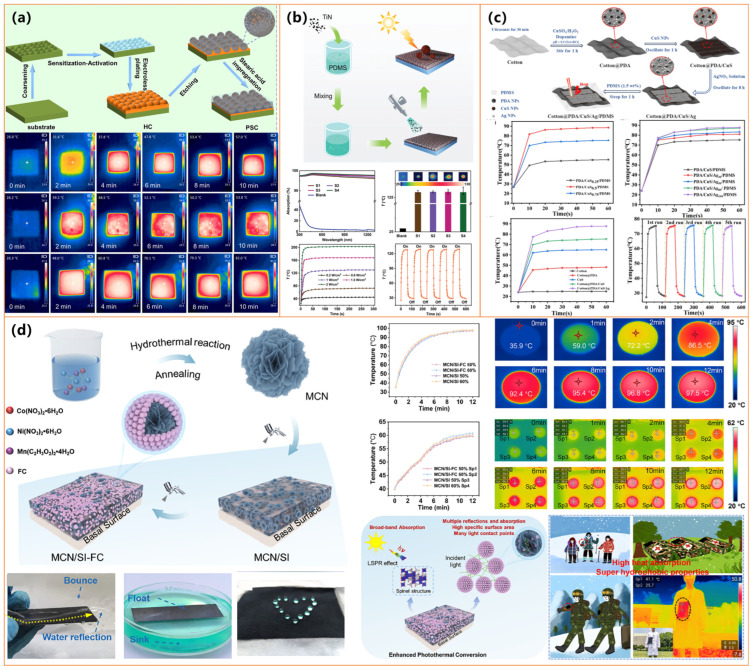
(**a**) Preparation process of PSC and infrared camera images of the substrate, HC, and PSC [113]; (**b**) preparation and light absorption and photothermal performance of TiN coatings [22]; (**c**) preparation and photothermal curves of Cotton@PDA/CuS/Ag/PDMS and other cotton fabrics [120]; (**d**) preparation, photothermal properties, and applications of MCN/SI and MCN/SI-FC coatings [117].

Metallic compounds play a crucial role in photothermal superhydrophobic coatings, offering distinct advantages and diverse functionalities. Primarily including metal oxides (e.g., Fe_3_O_4_, Ti_2_O_3_), nitrides (e.g., TiN), and copper-based materials (e.g., Cu_2−x_S, CuO, Cu_2_O), these compounds exhibit strong light absorption, excellent thermal stability, and high mechanical strength [22,113,114,115,116,117,118,119,120]. Table 2 presents the details of recent studies on metallic-compound-based photothermal superhydrophobic surfaces. Their ability to absorb visible and near-infrared light enables efficient photothermal conversion, while superior thermal conductivity ensures uniform heat distribution. Additionally, their durability and abrasion resistance make them well-suited for extreme environments. Current research focuses on cost-effective alternatives, optimizing nanostructures for enhanced photothermal efficiency and integrating them with conductive polymers or carbon-based compounds for multifunctionality. As shown in Figure 10, future advancements will emphasize cost reduction, fluorine-free, environmentally friendly formulations, and multi-responsive functionalities to meet growing demands in anti-icing, anti-fouling, solar energy harvesting, and next-generation materials [22,113,114,115,116,117,118,119,120].

Wei et al. [113] developed a fluorine-free CuO-based coating using a chemical deposition and etching process, achieving rapid heating (82 °C in 10 min) under irradiation, significantly accelerating ice melting while maintaining strong durability, as shown in Figure 10a. Similarly, Lei et al. [114] designed a triple-layer Fe_3_O_4_-based coating, integrating PDMS for protection and hierarchical nanoparticles for enhanced solar absorption. This structure exhibited exceptional resistance to acidic and alkaline conditions, making it suitable for aerospace and power infrastructure applications. Zhang et al. [115] also applied plasma electrolytic oxidation (PEO) technology to fabricate a photothermal superhydrophobic coating on AZ31 magnesium alloy, incorporating Mg_1−x_CuₓO semiconductor phases for efficient solar absorption and ice prevention.

A key advantage of these coatings is their ability to achieve rapid photothermal heating under sunlight, significantly enhancing deicing efficiency. Wang et al. [22] demonstrated that a plasmonic TiN-embedded PDMS coating could reach 130 °C in just 90 s, leveraging the plasmon-enhanced photothermal effect to prevent ice adhesion, as shown in Figure 10b. Hou et al. [116] introduced microencapsulated phase-change materials (MPCMs) with Cu_2_O/Cu_2−x_S to combine photothermal conversion with thermal storage, achieving a conversion efficiency of 96.1% and stable thermal performance even after 200 cycles. Meanwhile, Zhou et al. [117] developed a cost-effective PVDF-based coating incorporating micro- or nanostructured metal oxides (MCN), reaching 97.5 °C under irradiation, demonstrating scalability and adaptability for outdoor applications, as shown in Figure 10d. In addition, Zheng et al. [118] and Wang et al. (Figure 10c) [120] employed similar surface modification methods to prepare CuS-based composite coatings on cotton fabrics, achieving consistency in properties such as superhydrophobicity and photothermal performance. However, they differ in functional emphasis: Zheng et al. [118] focused on reversible conductivity and UV resistance, demonstrating applications in smart textiles and oil-water separation, whereas Wang et al. [120] incorporated silver nanoparticles and polydopamine (PDA) to endow the fabric with omnipotent antibacterial activity, effective even without light exposure, making it more suitable for medical and hygienic uses.

Beyond anti-icing properties, these metallic-compound-based coatings exhibit strong mechanical durability and environmental resistance, maintaining their functionality even after prolonged exposure to abrasion, water impact, and chemical corrosion. Wang et al. [120] fabricated a CuS-decorated cotton textile with PDMS treatment, achieving efficient photothermal conversion and making it suitable for extreme weather protective clothing. Expanding on the textile-based approach, Wang et al. integrated PDA/CuS/Ag/PDMS onto cotton fabrics, incorporating antibacterial functionalities with a 99.9% bactericidal rate under NIR exposure, making it a promising material for medical protective textiles, as shown in Figure 10c. Similarly, Luo et al. [119] employed oxygen plasma treatment and multiple spray-coating steps to develop a superhydrophobic polypropylene (PP) fabric with AgNPs and Fe_3_O_4_ nanoparticles, ensuring mechanical flexibility, photothermal efficiency, and self-cleaning properties. Future advancements in photothermal superhydrophobic coatings will focus on cost-effective fabrication, multi-functional integration, and long-term durability under extreme conditions. Zhang et al. [115] explored a near-infrared laser-mediated ZnO/CuO-based coating, further enhancing its anti-icing and antimicrobial performance.

In conclusion, metals and metallic compounds hold significant potential in the development of photothermal superhydrophobic coatings for anti-icing and deicing applications. Through advanced processing techniques and integration with multifunctional materials, metal-based coatings can achieve superior photothermal and superhydrophobic properties, while metallic compounds provide additional functionalities and material versatility. Future advancements will prioritize cost-effectiveness, durability, and environmental sustainability by exploring low-cost alternatives (e.g., black ceramics, Mn-Co-Ni-O), optimizing nanostructured architectures, and integrating multifunctional composites with polymers and carbon-based materials. Additionally, developing fluorine-free, eco-friendly coatings, lightweight and flexible materials, and multi-responsive functionalities will also enable adaptive deicing solutions. Progress in scalable, high-performance coatings will drive widespread adoption across critical sectors such as aviation, infrastructure, and smart textiles, offering innovative solutions for extreme environmental conditions. The following subsection will further explore the application prospects of metal compounds alongside non-metallic materials, expanding the scope of photothermal superhydrophobic coatings.

### 3.2. Carbon-Based Photothermal Superhydrophobic Materials

#### 3.2.1. Carbon Nanotube

Carbon-based materials, including carbon nanotubes (CNTs) [24,99,121,122,123,124,125], graphene [127,128,129], and carbonized biomass [130,131,132,133,134], are pivotal in developing photothermal superhydrophobic surfaces due to their exceptional light absorption, superior thermal conductivity, and robust mechanical properties. These materials exhibit broad-spectrum solar absorption, typically exceeding 90%, enabling efficient photothermal conversion and rapid surface heating—key attributes for effective anti-icing and deicing [24,99,121,122,123,124,125,126,127,128,129,130,131,132,133,134]. Their inherently low surface energy and ability to form hierarchical micro/nanostructures contribute to outstanding water repellency (WCA > 150°), minimizing water adhesion and significantly reducing ice formation [24,99,121,122,123,124,125,126,127,128,129,130,131,132,133,134]. Beyond their hydrophobicity and photothermal capabilities, carbon-based materials facilitate the fabrication of durable, self-healing, and flexible coatings, maintaining their functional integrity even under abrasion, extreme temperatures, and corrosive environments [24,99,121,122,123,124,125,126,127,128,129,130,131,132,133,134]. Furthermore, their compatibility with diverse fabrication techniques—including spray-coating, laser-induced graphitization, micro-arc oxidation, and template-assisted deposition—enhances their versatility for large-scale applications [24,99,121,122,123,124,125,126,127,128,129,130,131,132,133,134]. As a result, these materials are auspicious for deployment in aerospace, power grids, transportation, and outdoor infrastructure, where reliable anti-icing and deicing solutions are critical [24,99,121,122,123,124,125,126,127,128,129,130,131,132,133,134]. Table 3 presents the details of recent studies on carbon-based photothermal superhydrophobic surfaces. The specific research content will be elaborated in subsequent sections.

Carbon nanotubes (CNTs) have emerged as up-and-coming materials for fabricating photothermal superhydrophobic surfaces due to their unique structural and functional properties [22]. Their high aspect ratio, exceptional electrical and thermal conductivity, and intense light absorption can enable efficient photothermal conversion, allowing for rapid surface heating, which is crucial for anti-icing and deicing applications [99]. The interconnected CNT network facilitates uniform heat distribution, enhancing thermal performance across coated surfaces. Additionally, their low surface energy promotes the formation of superhydrophobic coatings with high water contact angles (WCA > 150°) and low adhesion, effectively preventing water accumulation and ice formation [24,99,121,122,123,124,125]. The mechanical robustness and flexibility of CNT-based coatings further contribute to their durability, enabling resistance to abrasion, bending, and extreme environmental conditions. Moreover, CNTs can be functionalized with various chemical modifications (e.g., fluorosilane modification) to improve chemical resistance and self-healing properties, thereby extending their operational lifespan [121,122,123,124,125]. Their compatibility with scalable fabrication techniques, including spray-coating, dip-coating, and electrostatic deposition, makes them highly suitable for large-scale implementation in anti-icing and deicing applications [24,99,121,122,123,124,125].

**Figure 11:**
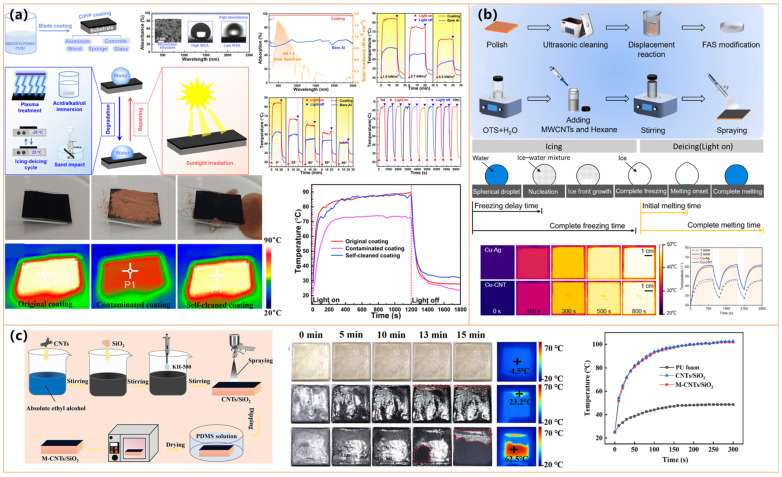
(**a**) Preparation and photothermal properties of MWCNTs/PVDF/PDMS (C/P/P) coating for anti-icing/deicing [122]; (**b**) preparation, icing mechanism, and temperature changes in Cu–Ag and Cu-CNT surfaces [99]; (**c**) fabrication of M−CNTs/SiO_2_ and photothermal properties of PU foam, CNTs/SiO_2_ coating, and M−CNTs/SiO_2_ coating [125].

Several studies have demonstrated the effectiveness of integrating carbon nanotubes (CNTs) with various substrates to enhance photothermal performance and mechanical durability. Wu et al. [121] developed a PMMA@MWCNTs membrane that rapidly reached 80.9 °C under sunlight, significantly delaying freezing (1175 s) and enabling rapid deicing (160 s). Similarly, Li et al. [122] fabricated a PDMS/PVDF-based coating incorporating PDA/ODA-modified MWCNTs, achieving high photothermal efficiency (~65 °C) and excellent self-healing properties, making it resistant to chemical and mechanical wear, as shown in Figure 11a.

Further advancements in CNT-based coatings have been reported by Xie et al. [24], who utilized electrochemical deposition and silanization to achieve an impressive surface temperature of 90 °C, extending freezing delay up to 3600 s at −15 °C and melting ice within 94 s. Zhang et al. [123] introduced a CNTs-SiO_2_/epoxy composite that maintained robust mechanical stability under friction and peeling tests while exhibiting intense photothermal heating for efficient deicing. Sun et al. [99] explored CNT–metal hybridization by incorporating Cu-CNT composites, as shown in Figure 11b. This facilitated ice detachment before completely melting, reducing ice adhesion and ensuring long-term performance across multiple icing/deicing cycles.

Expanding on multi-layered coatings, Peng et al. [124] designed a photothermal superhydrophobic coating using antifreeze protein (AFP)-modified emulsified asphalt. This coating ensures sustained anti-icing effects even after surface wear, making it particularly suitable for road applications. Meanwhile, Guo et al. [125] employed a PU foam-based hierarchical structure with CNTs and SiO_2_ nanoparticles, achieving the highest reported photothermal heating (101.9 °C) and rapid surface warming (62.5 °C in 15 min at −20 °C), enabling effective ice prevention and removal, as shown in Figure 11c.

Overall, while all studies emphasize the synergy between superhydrophobicity and photothermal conversion, key differences lie in fabrication methods, durability, and additional functionalities. Some materials, such as those by Li et al. [122] and Peng et al. [124], focus on self-healing and long-term resilience, while others, like those developed by Guo et al. [125], prioritize extreme photothermal efficiency. These technologies present promising prospects for large-scale anti-icing applications in aerospace, transportation, and infrastructure, paving the way for energy-efficient and sustainable deicing solutions.

#### 3.2.2. Graphene

Graphene-based materials, including graphene oxide (GO), reduced graphene oxide (rGO), and laser-induced graphene (LIG), have garnered significant attention in the development of photothermal superhydrophobic surfaces due to their outstanding thermal conductivity, broad-spectrum light absorption (~98%), and superior mechanical robustness [127,128,129]. These properties enable graphene-based coatings to achieve rapid and efficient photothermal conversion, which is critical for anti-icing and deicing applications [127,128,129]. The unique two-dimensional structure and high surface area of graphene facilitate the formation of hierarchical micro/nanostructures, enhancing water repellency and reducing ice adhesion [127,128,129]. Furthermore, graphene-based coatings exhibit remarkable durability, maintaining superhydrophobicity even under mechanical abrasion, extreme temperatures, and chemical exposure [127,128,129]. Functional modifications, such as fluorination or hybridization with silica nanoparticles, further enhance self-cleaning properties and corrosion resistance [127,128,129]. Additionally, graphene-based materials are highly adaptable to various scalable fabrication techniques, including laser-induced graphitization, spray-coating, and micro-arc oxidation. They are well suited for large-scale industrial applications in aerospace, power transmission, and transportation [127,128,129].

**Figure 12:**
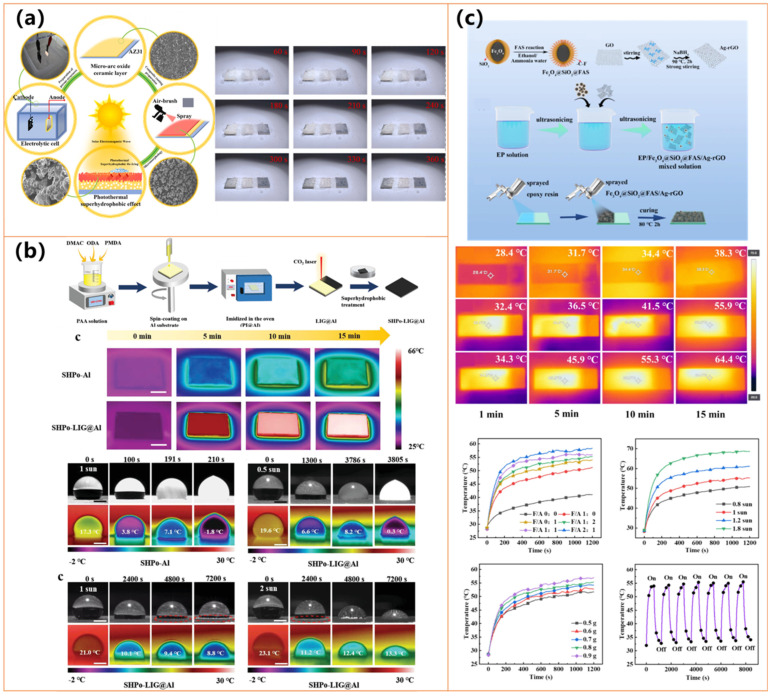
(**a**) Preparation process of photothermal superhydrophobic composite anti-freeze coating and photothermal curves of AZ31, AZ31-MAO, and photothermal superhydrophobic composite coating [126]; (**b**) preparation process of SHPo-Al and SHPo-LIG@Al surfaces and photothermal anti-icing abilities of SHPo-Al and SHPo-LIG@Al under illumination [129]; (**c**) preparation and photothermal performance of EP/Fe_3_O_4_@SiO_2_@FAS/Ag-rGO coatings under 1-sun illumination [128].

Studies by Zhang et al. [126] (shown in Figure 12a) and Li et al. [127] demonstrated that hybridizing graphene with silica nanoparticles (SiO_2_) or fluorinated SiO_2_ further enhances water repellency and reduces ice adhesion. These coatings exhibit remarkable superhydrophobicity (WCA > 160°) and superior durability, maintaining their properties under mechanical abrasion, chemical exposure, and extreme temperatures. Incorporating additional functional materials, such as titanium carbide (TiC) and silicone resin (MSR), as explored by Li et al. [127], enables self-healing capabilities and improved corrosion resistance, making these coatings viable for long-term industrial applications.

The photothermal performance of these coatings plays a crucial role in their anti-icing efficiency. Zhang et al. [128] reported that a graphene–SiO_2_ coating reached a stable surface temperature of 49.56 °C under solar irradiation, effectively minimizing ice formation. Similarly, Li et al. [127] demonstrated that rGO-based coatings rapidly heated under sunlight, facilitating efficient deicing and extended freezing delay. Notably, Wang et al. [129] developed a laser-induced graphene (LIG) coating that achieved a significantly higher surface temperature of 65 °C under one-sun irradiation, ultimately preventing ice formation under sufficient solar exposure, as shown in Figure 12b. These findings highlight the potential of graphene-based materials for passive and active anti-icing applications by leveraging their superior photothermal conversion properties.

In addition to graphene-based coatings, researchers have further explored the synergistic integration of metal oxides and noble metals to enhance anti-icing performance. Zhang et al. [128] introduced a Fe_3_O_4_@SiO_2_@Ag-rGO coating, which achieved a surface temperature of 58.8 °C and extended the freezing time up to 1498 s at −20 °C, significantly outperforming conventional coatings, as shown in Figure 12c. The combination of Fe_3_O_4_ and Ag nanoparticles with graphene-based structures enhances light absorption and heat conversion efficiency, making these coatings highly effective for icy environments. Additionally, the coatings maintain strong mechanical and chemical stability, ensuring long-term anti-icing performance. Overall, recent advancements in photothermal superhydrophobic coatings emphasize the importance of hierarchical micro/nanostructures, efficient heat conversion, and material durability.

#### 3.2.3. Other Materials

In addition to CNTs and graphene, other carbon-based nano- and micromaterials have emerged as effective candidates for photothermal superhydrophobic coatings due to their excellent light absorption, thermal insulation, and durability [130,131,132,133,134]. Their inherently porous structures enhance light trapping, thereby improving photothermal conversion efficiency and minimizing heat dissipation, which is crucial for anti-icing and deicing applications [130,131,132,133,134]. These materials also exhibit low surface energy, with water contact angles exceeding 150°, ensuring exceptional water repellency and reduced ice adhesion. Furthermore, their strong resistance to mechanical wear, extreme temperatures, and harsh environmental conditions enhances their long-term stability [130,131,132,133,134]. Functional modifications, such as fluorination and silica hybridization, further improve self-cleaning properties and corrosion resistance [130,131,132,133,134]. Their compatibility with scalable fabrication techniques, including spray-coating and dip-coating, makes them highly suitable for large-scale transportation, infrastructure, and energy systems applications [130,131,132,133,134].

**Figure 13:**
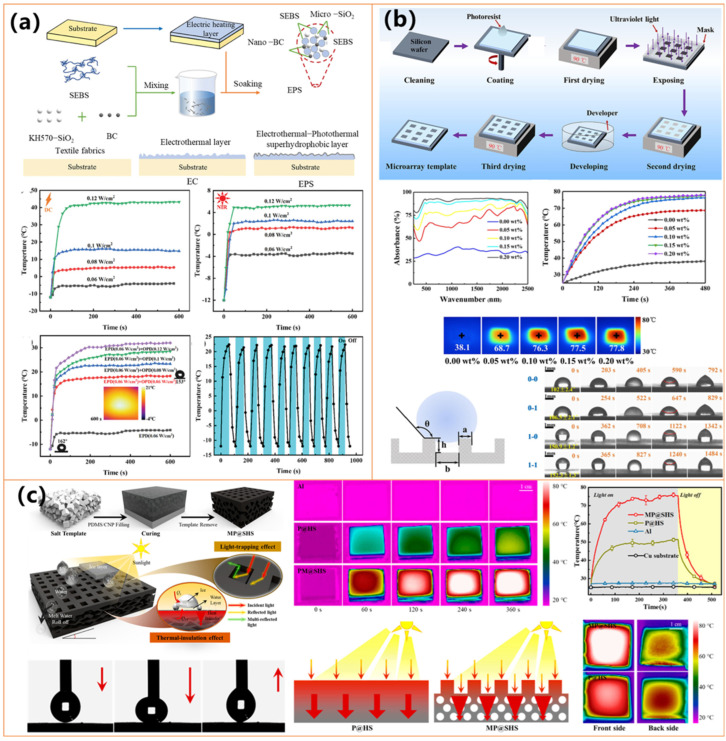
(**a**) Preparation and electrothermal and photothermal properties of EPS [130]; (**b**) fabrication and photothermal conversion properties of microarrays with different types of carbon black [133]; (**c**) fabrication and photothermal properties of MP@SHS [134].

Several studies have explored different approaches to optimizing these coatings for enhanced anti-icing performance. Deng et al. [130] introduced an electro-photothermal superhydrophobic (EPS) nanocomposite combining biochar, KH570-SiO_2_, and SEBS, which exhibited both photothermal and electrothermal heating, maintaining surface temperatures above 0 °C under sunlight and reducing deicing time by up to fivefold (as shown in Figure 13a). Zhang et al. [131] developed a sustainable photothermal coating using carbonized biomass and hydrophobic silica nanoparticles, which achieved rapid solar heating and excellent mechanical robustness, making it suitable for outdoor applications. Meanwhile, Wu et al. [132] designed a candle-soot-derived superhydrophobic surface with a silica shell and PDMS modification, demonstrating exceptional icephobicity even at −50 °C and rapid deicing within 300 s under sunlight exposure.

Further innovations in fabrication techniques have improved the scalability and practicality of these coatings. Guo et al. [133] utilized a flexible, spray-coated photothermal superhydrophobic material incorporating carbon-based nanomaterials and polymeric binders, offering strong mechanical durability and adaptability for large-scale applications in transportation and infrastructure (as shown in Figure 13b). Xie et al. [134] (as shown in Figure 13c) introduced a salt-templated microporous superhydrophobic coating that exhibited 98.3% light absorption and enhanced thermal insulation, leading to ice formation delays of up to 648.1 s at −20 °C and rapid ice melting within 129.1 s. The microporous architecture effectively reduced heat dissipation, significantly improving anti-icing and deicing efficiency compared to conventional coatings.

These findings highlight the versatility and effectiveness of carbon-based photothermal superhydrophobic coatings in mitigating ice formation across diverse environmental conditions. While all studies confirm their high superhydrophobicity and photothermal performance, material selection and structural design variations contribute to unique enhancements, such as electrothermal integration, sustainable biomass utilization, and flexible spray-coating techniques. Together, these innovations pave the way for scalable, durable, and high-performance anti-icing solutions in aviation, transportation, and renewable energy systems, where ice accumulation remains a significant challenge.

In conclusion, carbon-based photothermal superhydrophobic coatings have demonstrated exceptional anti-icing, deicing, and durability properties, making them highly suitable for applications in aerospace, power grids, transportation, and outdoor environments. Carbon nanotubes (CNTs) contribute high conductivity and mechanical strength; graphene enhances thermal performance and structural robustness, while other carbon-based materials, such as biochar and carbonized biomass, offer cost-effective and sustainable alternatives. Integrating these materials with hierarchical structures, polymer matrices, and functional modifications (self-healing properties, electrothermal integration, and hybridization with metal nanoparticles) advances anti-icing and self-cleaning technologies. Future research will optimize these coatings’ scalability, environmental adaptability, and multifunctionality, emphasizing fluorine-free, eco-friendly formulations and enhanced electrical conductivity. As a result, carbon-based coatings are poised to play a critical role in next-generation deicing technologies, providing durable, energy-efficient, and sustainable solutions for extreme environmental conditions across multiple industrial sectors.

### 3.3. Polymer-Based Photothermal Superhydrophobic Materials

Polymers such as polypyrrole (PPy), polydopamine (PDA), polydimethylsiloxane (PDMS), and fluorine-free silanes play a crucial role in developing photothermal superhydrophobic coatings due to their unique mechanical durability, environmental sustainability, and tunable surface properties, as well as their lightweight nature and cost-effectiveness [135,136,137,138,139,140,141,142]. These materials enable coatings to achieve high water contact angles (>150°) and low sliding angles, ensuring excellent water repellency and self-cleaning functionality. Table 4 presents the details of recent studies on polymer-based photothermal superhydrophobic surfaces. Additionally, conductive polymers (PPy, PDA), in combination with metal nanoparticles (Ag NPs, Fe_3_O_4_), facilitate efficient photothermal conversion, allowing for rapid heating under solar irradiation and significantly improving anti-icing and deicing performance [135,136,137,138,139,140,141,142]. Organic coatings also exhibit superior mechanical robustness, UV resistance, and chemical stability, making them highly suitable for long-term outdoor applications. Furthermore, developing fluorine-free, biopolymer-based coatings (e.g., lignin-derived materials) enhances environmental sustainability while maintaining high-performance properties [135,136,137,138,139,140,141,142]. With ongoing advancements in hybrid materials, large-scale fabrication techniques, and innovative adaptive coatings, organic materials are expected to dominate the next generation of photothermal superhydrophobic coatings for anti-icing and deicing applications.

**Figure 14:**
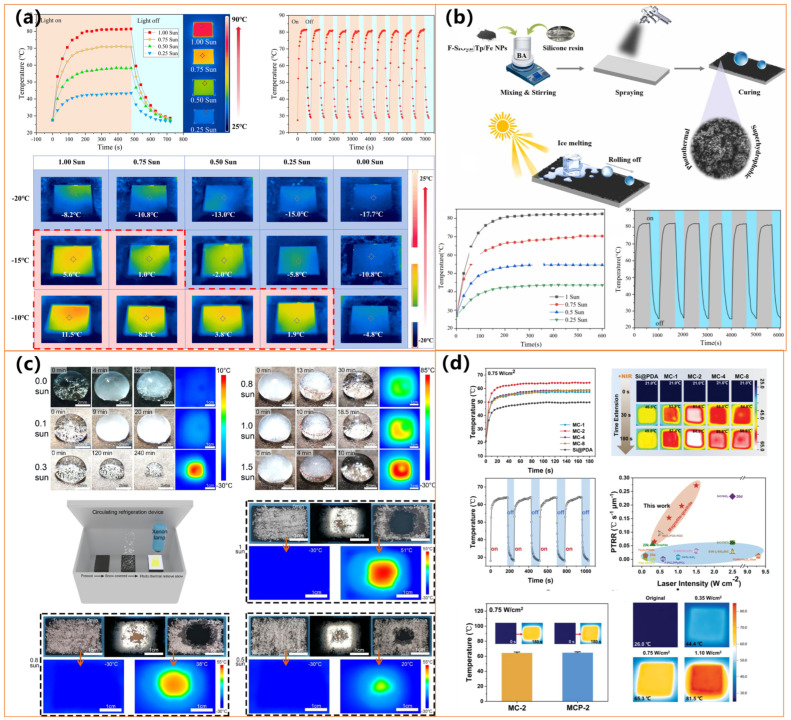
(**a**) Photothermal properties of FCF−BP/SiO_2_ coating [135]; (**b**) preparation and photothermal properties of superhydrophobic F−SiO_2_ @Tp/Fe coatings [138]; (**c**) photothermal properties of PSP−SPONGE [139]; (**d**) photothermal −roperties of MCs and Si@PDA [131].

Studies by Yuan et al. [135] (as shown in Figure 14a) and Ma et al. highlight the potential of hybrid organic coatings, such as fluorocarbon resin-based coatings (FCF-BP/SiO_2_) and lignin micro-nanosphere (LMNS)-based coatings, both of which achieve superhydrophobicity (WCA > 155°) while demonstrating high photothermal conversion efficiencies, enabling rapid heating and effective deicing. Additionally, Xiong et al. [136] and Xie et al. [137] have further advanced PPy-based coatings by incorporating attapulgite nanorods and polymer-modified PET fabrics, significantly improving thermal stability and achieving 70–91 °C temperatures under simulated sunlight.

Researchers have incorporated metal nanoparticles and oxides into organic coatings to enhance photothermal efficiency and mechanical durability. Jiang et al. [138] developed an iron-based multifunctional coating (F-SiO_2_@Tp/Fe, as shown in Figure 14b), which combines superhydrophobicity (WCA = 159°) with efficient sunlight absorption, delaying ice formation more than threefold at −25 °C. Similarly, Yu et al. [139] designed a Fe_3_O_4_ nanoparticle and PDA-modified polyurethane sponge (PSP-SPONGE), which maintained anti-icing properties for 120 min at −30 °C with minimal sunlight exposure, as shown in Figure 14c. The incorporation of silver nanoparticles has also proven effective, as demonstrated by Wu et al. [140], who fabricated a PDA@PEI@GA@Ag@PDMS-coated fabric that achieved a surface temperature of 70.4 °C under simulated sunlight, ensuring rapid deicing. Ding et al. [142] further developed a dual-layer nanofibrous membrane incorporating Ag NPs and PDMS, effectively preventing water penetration while enabling rapid heat transfer for anti-icing applications. In addition to iron-based materials, Zhang et al. [131] (as shown in Figure 14d) introduced a virus-like iron oxide mineral coating with hierarchical micro/nanostructures, exhibiting excellent superhydrophobicity and superior photothermal performance under near-infrared (NIR) irradiation. The coating rapidly increased in temperature, facilitating efficient frost melting within 20 s and complete ice removal within 600 s, demonstrating its high potential for extreme cold environments.

Beyond performance optimization, researchers have focused on enhancing long-term durability and environmental sustainability. Yuan et al. [135] demonstrated that their fluorocarbon resin-based coating retained water repellency after 300 abrasion cycles and 100 tape-peeling tests. At the same time, Ma et al. [141] reported that their LMNS-based coating withstood 320 cm of abrasion and 210 tape-peeling cycles. Similarly, Xiong et al. [136] and Xie et al. [137] showcased PPy-based coatings with excellent stability under extreme conditions, including high temperatures (up to 180 °C) and prolonged UV exposure. Meanwhile, environmentally friendly approaches, such as fluorine-free biopolymer-based coatings developed by Ma et al. [141], provide sustainable alternatives without compromising anti-icing performance, marking a shift toward green material development.

Organic-based photothermal superhydrophobic coatings have demonstrated outstanding effectiveness in mitigating ice formation and enabling rapid deicing across diverse environmental conditions. Integrating organic polymers with metal-based nanostructures enhances photothermal efficiency, while hybrid material strategies improve mechanical robustness and long-term durability. As research advances, developing innovative, self-healing, and scalable coatings—such as Yu et al.’s PSP-SPONGE—will be crucial for real-world applications in aerospace, marine, and infrastructure settings [139]. Future efforts will focus on optimizing large-scale fabrication techniques, developing fluorine-free eco-friendly formulations, and incorporating multifunctional properties such as antibacterial activity and adaptive responsiveness. With the continuous integration of conductive polymers, bio-based materials, and hybrid metal–organic composites, organic-based coatings are poised to drive the next generation of sustainable and high-performance anti-icing and deicing technologies.

Overall, from the current research on photothermal superhydrophobic coatings, it is evident that the construction of such coatings often involves the combination of multiple material types rather than relying on a single type of material. For instance, in the study by Zhang et al. [128] on Fe_3_O_4_@SiO_2_@Ag-rGO coatings, a mixture of metal, metal compound, and carbon-based materials was employed. The integration of diverse materials not only enhances the superhydrophobic and photothermal performance of the coatings but also expands their functional versatility, offering broader application prospects beyond those of conventional superhydrophobic or photothermal coatings.

## 4. Application Scenarios of Photothermal Superhydrophobic Surfaces for Anti-Icing/Deicing

**Figure 15:**
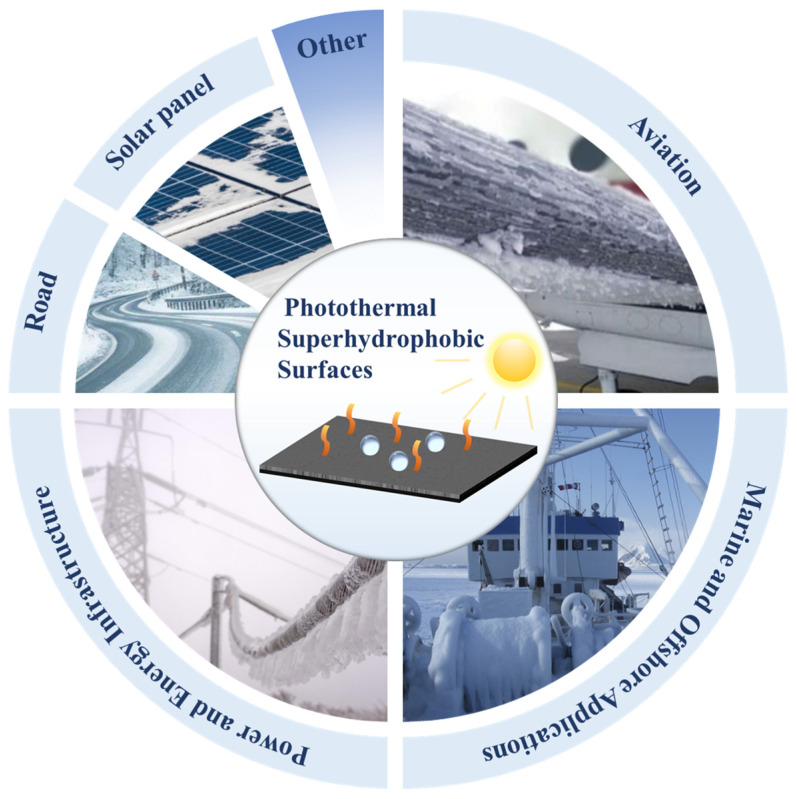
Application scenarios of photothermal superhydrophobic surfaces for anti-icing/deicing.

Photothermal superhydrophobic coatings, combining light-driven thermal conversion with water-repellent nanostructures, are emerging as a critical solution to address icing challenges across diverse sectors. Researchers are advancing their applications in aviation, energy infrastructure, marine systems, etc. Superhydrophobic surfaces can magnify the water contact angle, while photothermal agents, such as carbon nanomaterials and metal oxides, can passively harness solar energy to generate heat. This synergy inhibits ice formation, reduces adhesion, and enables energy-efficient deicing (Figure 15).

### 4.1. Aviation

Aircraft operating at high altitudes and UAVs deployed in cold environments face severe icing issues that compromise flight safety, stability, and operational efficiency. For aircraft, ice accumulation on wings and fuselage increases aerodynamic drag, elevates fuel consumption, and raises risks of mechanical failure [122,135]. These materials integrate superhydrophobicity and photothermal conversion: the former minimizes water adhesion, while the latter converts solar energy into localized heat. This combined effect elevates surface temperatures, inhibits ice nucleation, and weakens ice adhesion, significantly lowering long-term maintenance costs [122,141].

For UAVs, lightweight photothermal coatings are critical, especially for their solar-powered or autonomous systems. Ice buildup on propellers and airframes disrupts aerodynamics and sensor functionality, jeopardizing mission success. By leveraging sunlight as an energy source, these coatings passively prevent icing without requiring additional power input, ensuring stable flight performance in subzero conditions [107]. The technology is particularly advantageous for long-endurance UAVs and extreme environments, where minimizing weight and energy consumption is paramount.

### 4.2. Power and Energy Infrastructure

Ice accumulation on high-voltage power lines and wind turbine blades poses significant risks to energy reliability and operational efficiency in cold climates. Photothermal superhydrophobic coatings offer a passive solution by utilizing sunlight to mitigate icing. These coatings absorb solar radiation for power lines to elevate surface temperatures, reducing ice adhesion and minimizing the need for manual deicing. This approach enhances grid stability, particularly in remote or high-altitude regions, while lowering maintenance costs [98,144]. Similarly, on wind turbine blades, the coatings convert absorbed light into heat, preventing ice formation that degrades aerodynamic performance and disrupts energy production. By maintaining ice-free surfaces, the technology ensures consistent power generation during winter and reduces operational downtime [24,145]. Both applications demonstrate a cost-effective and sustainable strategy to address icing challenges in critical energy infrastructure, improving resilience without relying on chemical deicing agents or energy-intensive systems.

### 4.3. Marine and Offshore Applications

Marine vessels and offshore infrastructure operating in polar regions or winter seas face persistent icing risks that compromise safety, navigation, and operational continuity. Photothermal superhydrophobic coatings provide a unified strategy to address ice accumulation across these diverse maritime environments.

For maritime vessels such as icebreakers, fishing boats, and polar research ships, ice formation on exposed surfaces, including decks, masts, and navigation sensors, impairs stability and equipment functionality. Coatings applied to these areas leverage solar energy to passively elevate surface temperatures, preventing ice adhesion and minimizing the need for hazardous manual deicing. This enhances crew safety and ensures uninterrupted navigation in icy waters, particularly critical for vessels traversing high-latitude routes where rapid ice accretion can obstruct radar and communication systems [98,146].

Subzero temperatures in offshore oil and gas platforms lead to ice buildup on pipelines, sensor arrays, and safety-critical equipment, leading to operational delays and mechanical failures. Photothermal coatings on these structures inhibit ice nucleation through a combination of superhydrophobic surface properties and localized solar-driven heating. By maintaining ice-free conditions on valves, antennas, and monitoring instruments, the coatings reduce unplanned maintenance interventions and mitigate risks of ice-induced corrosion or sensor drift. This ensures reliable data collection and operational efficiency for offshore installations, even during prolonged cold spells, while extending the service life of exposed infrastructure [147,148].

### 4.4. Other Application Scenarios

Icy road surfaces pose severe safety risks by reducing vehicle traction and increasing accident rates, while traditional deicing methods like salt spreading exacerbate infrastructure corrosion and environmental harm. Photothermal superhydrophobic coatings offer a sustainable alternative by harnessing sunlight to generate heat, accelerating ice melting on roads. The coatings’ superhydrophobic properties simultaneously repel melted water, preventing refreezing and maintaining transparent surfaces. This dual functionality reduces reliance on ecologically damaging chemical deicers and minimizes long-term maintenance costs, particularly in regions with frequent winter storms. Integrating these coatings into asphalt or concrete allows transportation authorities to enhance road safety without compromising environmental integrity, addressing immediate hazards and systemic sustainability challenges [143,149].

Similarly, snow and ice accumulation on solar panels drastically diminishes energy output in cold climates by blocking sunlight absorption. Photothermal superhydrophobic coatings applied to panel surfaces enable passive ice and snow removal through solar-driven heating. The absorbed light converts into thermal energy, melting interfacial ice layers, while the low surface energy of the coating promotes the gravitational shedding of snow. This self-clearing mechanism ensures consistent power generation efficiency even during heavy snowfall, eliminating the need for manual cleaning or energy-intensive heating systems. For solar farms in snowy regions, the technology not only stabilizes energy production but also reduces operational interruptions, supporting the economic feasibility of solar energy in winter-prone areas. Both road and solar applications exemplify how photothermal coatings bridge infrastructure resilience with ecological stewardship, leveraging natural energy to counteract winter-related inefficiencies [150].

In addition, the photothermal superhydrophobic method exhibits promising application prospects in areas such as building facades, rooftops, and polar equipment. Through advanced materials science, this methodology is anticipated to catalyze transformative innovations in eco-friendly ice-resistant systems.

## 5. Challenges of Photothermal Superhydrophobic Surfaces for Anti-Icing/Deicing Applications

Photothermal superhydrophobic surfaces have emerged as a promising solution for ice management. They combine passive anti-icing/deicing through water repellency with active solar-driven deicing by converting light into heat. However, despite their functional advantages, several intrinsic limitations hinder their large-scale and long-term application, particularly under harsh environmental conditions.

A primary limitation stems from the reliance on direct solar irradiation. The photothermal mechanism requires unblocked exposure to sunlight to generate sufficient surface heating. Yet, in real-world scenarios where the surface is fully covered by frost or snow, light transmission is significantly reduced, severely restricting the photothermal effect [80,134,140]. Under such conditions, passive anti-icing properties may still function by delaying ice nucleation, but active deicing becomes largely ineffective without external energy input [116,140]. This issue is further compounded by the significant energy required to melt ice. With a latent heat of fusion of approximately 334 kJ/kg and an ice layer of 1 mm corresponding to ~1 kg/m^2^, the minimum energy demand reaches 334 kJ/m^2^. Under 1-sun irradiation (1000 W/m^2^), and assuming a realistic photothermal conversion efficiency of 50–85%, this translates to an estimated deicing time of 5–10 min, which aligns with experimental durations reported in prior studies [80,126,134,137,140]. However, the effectiveness drops dramatically in low-light or cloudy conditions. In addition to energy input, the thermal diffusion limitations of photothermal superhydrophobic surfaces also restrict their performance. Many of these coatings possess low intrinsic thermal conductivity, resulting in highly localized heating [116,140]. This can be problematic when melting larger or unevenly distributed ice layers. To mitigate this, some researchers have incorporated phase change materials, which absorb and store solar energy during illumination and release it gradually to prolong the deicing effect under intermittent light [116,140].

Another critical challenge is the mechanical durability of superhydrophobic surfaces [128,140]. Environmental factors such as sand erosion, rain impact, and repetitive freeze–thaw cycles can progressively damage the delicate surface of micro-/nanostructures, reducing hydrophobicity and diminishing photothermal efficiency [133,144]. Studies have reported noticeable degradation after 100 cycles of abrasion or tape peeling, underscoring the need for structural reinforcement [126,144]. In this context, it is essential to acknowledge that a performance trade-off often exists between photothermal efficiency and durability [128,140]. Highly efficient photothermal materials such as soot, MWCNTs, or PPy frequently rely on loosely bonded, high-surface-area structures that are vulnerable to wear [136,140,142]. Conversely, coatings reinforced with rigid binders or encapsulated in robust matrices offer excellent durability but may exhibit slower heating or lower light absorption [116,128]. Researchers have adopted various approaches to reconcile this contradiction, including hierarchical dual-scale surface designs, hybrid nanofillers, and self-healing polymers that enable dynamic repair under thermal stimulus [128,130,133,144]. These strategies aim to bridge the gap between functionality and resilience, moving closer to practical, long-lasting anti-icing/deicing solutions.

Some researchers have also proposed electro-photothermal hybrid systems that combine solar heating with Joule heating elements to ensure reliable performance in weak-light or snow-covered environments [126,130]. These systems can operate continuously and autonomously, making them well-suited for critical infrastructure such as power lines, wind turbines, and aircraft surfaces [130].

In conclusion, while photothermal superhydrophobic surfaces hold considerable promise for ice mitigation, their real-world application is still constrained by light dependence, energy demands, limited heat transfer, and surface wear challenges. Nevertheless, ongoing innovations in material design, structural reinforcement, and multi-mode thermal management are steadily improving the practicality and robustness of these coatings, paving the way toward durable, efficient, and all-weather ice control technologies.

## 6. Conclusions and Prospects

Photothermal superhydrophobic coatings, merging exceptional water repellency with efficient light-to-heat conversion, have emerged as transformative solutions for combating ice accumulation. This review emphasizes the research progress of advanced material systems, such as metal-based materials, carbon-based materials, and polymer composites, in enabling efficient anti-icing through surface treatment and performance optimization. Key advancements in these materials leverage intrinsic characteristics like high thermal conductivity, broad-spectrum light absorption, and chemical stability, achieved via structural and functional optimizations. These engineered materials exhibit robust adaptability in aviation, renewable energy infrastructure, marine systems, and transportation, providing energy-efficient and durable solutions tailored to extreme environmental challenges.

Despite their promising applications in anti-icing and deicing, challenges persist in achieving long-term durability under harsh conditions such as mechanical abrasion, extreme humidity, and UV exposure. Additionally, scalable fabrication processes require simplification to enhance industrial viability. Current methods often rely on multi-step procedures, which hinder reproducibility and industrial adoption. Future research should prioritize fluorine-free, eco-friendly formulations, self-healing mechanisms, and cost-effective fabrication techniques. Additionally, exploring their potential in emerging fields such as medical devices or smart textiles could further broaden their multifunctional utility. By overcoming these limitations, photothermal superhydrophobic systems could revolutionize anti-icing/deicing technologies while enhancing safety and sustainability in cold climates. Continued advancements in scalable production, environmental resistance, and multifunctional design will drive their adoption in aerospace, renewable energy, and smart infrastructure, laying the groundwork for next-generation ice-resistant and cross-domain applications.

## Figures and Tables

**Table 1:** Research on metal-based photothermal superhydrophobic surfaces.

Materials	Preparation Method	Water Contact Angle	Photothermal Performance	Anti-Icing/Deicing Ability	Durability& Other Features	Ref.
Copper mesh, Cu_x_S (CuS/Cu_2_S), stearic acid	Hydrothermal growth of Cu_x_S + SA	169°	Temp ↑ to 48.1 °C (808 nm laser)	Freezing delay: time extended to 27 min; Fast deicing	UV, Acid/base, Bending, Abrasion, Ultrasonic; Long-term storage; Corrosion protection; Oil-water separation	[100]
Cu foil, Cu-CAT-1 MOF, OTS	In situ MOF growth on Cu foil + OTS modification	155.8°	Rapid heating under xenon lamp; effective up to 480 s for deicing	Freezing delay: 870 s; Deicing: 480 s	Chemically stable in acid/base/salt solutions;	[102]
Cu@Cu_2_O foam, n-dodecanethiol, polydopamine	Oxidation + surface hydrophobic/hydrophilic functionalization	/	Photothermal conversion efficiency: 92%, Temp ↑ under 1 sun	/	Stable in continuous desalination,	[103]
Iron foam, CuO layer, PFDTES	Chemical etching + sintering + PFDTES modification	/	Temp ↑ to 55.8 °C (240 s under sunlight)	Freezing delay: 1220 s (from 221 s); Deicing in 251 s (0.2 W/cm^2^)	sandpaper abrasion (50 cycles); Water impact; Corrosion resistance	[104]
Cotton fabric, Ag NPs, PDMS	Dip-coating with Ag/PDMS in isopropanol, curing at 140 °C for 5 h	171.3°	Surface temperature increases under NIR; deicing under −20 °C	Photothermal deicing; Freezing and melting delay	Maintains WCA and UPF after washing (AATCC-61); Abrasion (Martindale, sandpaper); UV shielding (UPF tested); Antibacterial	[105]
Cotton, Ag/CdS, PDA, PFDT, or NDM thiols	PDA-assisted Ag/CdS deposition + thiol self-assembly	/	Electro-/photothermal self-healing under IR, xenon lamp, Joule heating	/	Stable: 18 accelerated wash cycles (≈90 home cycles); Thermal/photo-healing; Corrosion resistance (electrochemically tested); Photocatalytic Methylene Blue degradation; Antibacterial; Electrically conductive	[106]
MXene (Ti_3_C_2_Tₓ), Ag nanowires, PEDOT: PSS	Drop-casting + hydrophobic spray with fluorosilane	/	Photo-/electrothermal conversion for self-heating in cold/damp	Effective under low-temperature/high-humidity conditions	Strong adhesion via interlocking; Stable under humidity and cold; EMI shielding (31.5 dB @ 10 µm)	[107]
MXene (Ti_3_C_2_T_x_), Au NPs, SiO_2_@FAS, WPU	Two-step spray coating of MXene@Au-WPU + fSiO_2_	153°	photothermal deicing efficiency of 73.1%	Anti-icing time: 1053 s (−20 °C, RH 68%)	Stable high humidity, and multiple cycles; Corrosion resistance (pH 1–13)	[108]
Au/TiO_2_ NPs on PTFE, PDMS	Sol–gel synthesis + spin coating	/	Surface temp ↑ >25 °C (3-sun illumination)	Deicing under light (effective)	Stable during cyclic fogging and deicing; Low-cost, simple, low-noble-metal loading	[109]
Carbon steel, HDTMS	Laser ablation + HDTMS chemical modification	156°	Temp ↑ from ~13 °C to ~65 °C (1 sun, 8 min)	Freezing delay 2547 s; Thawing (60 s, 0.5 sun)	WCA > 150° after 2000 g sand impact or 400 tape peels; No fluorine; Black surface	[110]
Fe particles, PDMS, candle soot (CS)	Spin coating + magnetic particle deposition + candle soot deposition	154.5°	1 mm of ice melted (237 s, 1 sun)	Freezing: 4.7× longer; Deicing in 237 s	Stable after 320 abrasion cycles; Acid/base/NaCl, liquid nitrogen; Water impact; Self-healing; Magnetically attachable; Fluorine-free	[93]
Aluminium alloy, Zn/ZnSb dendrites, PDMS	Femtosecond laser + chemical treatment + PDMS coating	161.5°	ΔT = 48.5 °C (300 s, 1 sun)	Ice melted in <2 min	Durable after sand abrasion; Water impact; Tape peel; 3-month outdoor test; Absorptivity 97.3%, minimal surface change	[111]
Aluminium, FDTS (fluorinated silane)	Laser surface direct writing + thermal evaporation of FDTS	161.2°	Solar absorption > 94.5%, ΔT > 60 °C, rapid heating	Strong anti-icing; Defrosting at −30 °C	Stable (−80–200 °C); Mechanical/thermal/cold tests passed; Self-cleaning; Light trapping; Plasmon-enhanced heat conversion	[112]

**Table 2:** Research on metallic-compound-based photothermal superhydrophobic surfaces.

Materials	Preparation Method	Water Contact Angle	Photothermal Performance	Anti-Icing/Deicing Ability	Durability& Other Features	Ref.
GO@Fe_3_O_4_, PDMS, PF lubricant, CuO nano-grass on Cu	Etching Cu → CuO + lubricant infusion (PDMS/PF) with GO@Fe_3_O_4_	/	ΔT = 71.7 °C (infrared, 120 s)	Icing-delay: 269 s, −10 °C; Deicing: photothermal melting; Extremely low ice adhesion strength	Stable over 12 months; Self-healing; Anti-aging; Self-healing	[22]
Electroless-plated Cu, etched CuO	Plating + surface etching to form micro/nano CuO	/	ΔT = 82 °C (10 min, simulated light)	Deicing time: 1/3 of the bare substrate	Friction-/water-impact-stable; Alkali-resistant; Strong adhesion resistance; Self-cleaning; Antifouling; Fluorine-free	[113]
Fe_3_O_4_ (dual-size NPs), PDMS, Epoxy	Spray coating of Fe_3_O_4_@SiO_2_@FAS + PDMS + epoxy layer	/	ΔT = 14.4 °C/min; Efficiency = 62.38%;	Deicing in 790 s under sunlight, rate: ~1.96 kg/m^2^·h	pH 1–14, 20-day corrosion, 90 tape peels, 30 sand impacts;	[114]
Black ceramic (Mg_1−x_Cu_x_O), PDMS NPs	Plasma etching + black ceramic coating + PDMS vapor deposition	156°	Up to 69.4 °C (200 mW/cm^2^)	Photothermal active + superhydrophobic passive melting strategy	Validated long-term thermal and mechanical performance; MD simulation; Cassie–Wenzel transition optimization	[115]
3D Cu_2−x_S@Cu_2_O MPCM + OTS-based matrix	MPCM + hydrolysis + polycondensation with silane	/	Efficiency: 96.1%; Full-spectrum absorption	Efficient at low T, high humidity	200-cycle phase change with minimal thermal change; Phase change thermal storage + LSPR Cu_2−x_S optimization	[116]
MCN (Mn_x_CoNi_1−x_O_y_), PVDF, silicone resin	Solvothermal synthesis + two-step spray with silicone/PVDF	155.79°	Temp ↑ to 97.5 °C (1 kW/m^2^)	/	Excellent mechanical durability; Adheres to multiple substrates; Low-cost; Hierarchical micro/nanostructure	[117]
Cotton, CuS nanoflowers, PDMS	TA polymerization + CuS deposition + PDMS encapsulation	153.0 ± 0.4°	NIR- /unlight-responsive; Resistance drops (250 mW/cm^2^)	/	Maintains hydrophobicity after 40 sandpaper abrasion cycles; UV resistance; Oil–water separation	[118]
PP fabric, AgNPs, Fe_3_O_4_, PDMS	O_2_ plasma + AgNPs deposition + spray Fe_3_O_4_,/PDMS	/	Electro-photothermal effect; Joule-/light-responsive	/	Stable after bending, abrasion, and ultrasonic washing; EMI SE ≈ 56.1 dB (X-band); multi-responsive,	[119]
Cotton, PDA, CuS, AgNPs, PDMS	PDA/CuS/Ag deposition + PDMS encapsulation	/	Photothermal antibacterial (NIR and dark conditions); dual-light responsive	/	Stable after abrasion, bending, and ultrasonic washing; Omnipotent antibacterial; Self-cleaning,	[120]

**Table 3:** Research on carbon-based photothermal superhydrophobic surfaces.

Materials	Preparation Method	Water Contact Angle	Photothermal Performance	Anti-Icing/Deicing Ability	Durability& Other Features	Ref.
PMMA, FAS-modified MWCNTs	Breath figure method + spray-coating MWCNTs	157°	Surface T ↑ to 80.9 °C (120 s, 1 sun)	Freezing delay: 1175 s; Deicing: 160 s; Defrosting: 521 s	Stable after water/sand impact; Simple fabrication; Porous micro-nanostructures	[24]
Ag NPs on Cu (Cu–Ag), MWCNTs on Cu (Cu–CNT)	Cu–Ag: displacement deposition; Cu–CNT: OTS/MWCNTs spray	Cu–Ag: 162.1°; Cu–CNT: 158.3°	T ↑ Cu–Ag: 16.58 °C; Cu–CNT: 17.58 °C (100 s, 2 sun)	Freezing delay: 1336 s (Cu–Ag), 2926 s (Cu–CNT); Melt: ~473 s/421 s	Stable under cold/wet cycling; High repeatability; Scalable; Low-tox reagents	[99]
MWCNTs@PDA/ODA, PVDF, PDMS	Blade-coating on diverse substrates	>150°,	ΔT ≈ 65 °C (1 sun)	Superhydrophobicity restored after icing–thawing by sunlight	Resistant to plasma, oil, acid/base, sand, icing; Repairable by photothermal effect	[122]
CNTs-SiO_2_ hybrids, Epoxy	One-step spraying	159.3°	Rapid heating (Laser-induced)	Freezing delayed; Deicing: in seconds	Excellent chemical/ mechanical durability (tape-peel, friction); Hierarchical micro/nanostructure	[123]
AFP-modified emulsified asphalt, CNTs, SiC	Multilayer spray + curing	161°	Photothermal efficiency 50.94%	Freezing time doubled; Deicing under 2/4 kW/m^2^ NIR	Stable after 500-wheel rolls and 100 abrasion cycles; AFP core remains anti-icing after surface wear	[124]
CNTs, SiO_2_, PDMS, PU foam	Spraying + PDMS modification	157°	ΔT = 101.9 °C (1 kW/m^2^, 300 s)	Fast deicing	Stable after 10 freeze–thaw/20 friction cycles; Hierarchical structure	[125]
Graphene, SiO_2_, PDMS, Epoxy	Micro-arc oxidation on Mg alloy + spray coating	162.2°	Stable at 49.56 °C (2 sun)	Long-lasting deicing	Chemical/mechanical durability; 200 cm under 300g force	[126]
SiO_2_, GO, TiC, MSR	One-step spraying of MSR + modified additives	161.9°	/	Active deicing (808 nm NIR)	Stable after 60m of abrasion, kneading, corrosion, and plasma etch; Self-healing; Fluorine-free	[127]
Fe_3_O_4_@SiO_2_@FAS, Ag-rGO, Epoxy	Two-step spraying on epoxy base layer	153.8°	ΔT = 58.8 °C (1 sun)	Freezing delay: 1498 s; Deicing: 120 s, 40 mW/cm^2^	Stable under tape peel, friction, water, acid/base; Scalable	[128]
Laser-induced graphene (LIG) on PI/Al	Laser engraving + silanation on PI@Al	/	T ↑ to 65 °C (5 min, 1 sun); Absorption ≈98%	Retarded frost; Melting under 1–2 sun	Thermal, chemical, and corrosion stability; Scalable	[129]
EPS nanocomposite (Biochar, MWCNTs, SiO_2_, SEBS)	Blending + coating onto textiles	>150°	Maintaining T > 0 °C (−20 °C, PT/ET synergy)	Icing delayed 4×; Deicing time reduced 5× compared to PT only; All-weather anti-icing	UV, corrosion, and abrasion resistant; All-day effectiveness	[130]
Virus-like Fe_3_O_4_/Goethite nanostructures	Liquid-confined magnetite mineralization with PDA	/	Exceptional PT effect, comparable to carbon materials	Defrosting/deicing	Not quantified but implied via stability and reusability; Magnetic field assisted; Biocompatible; Scalable	[131]
Candle soot, SiO_2_ shell, PDMS brushes	Soot deposition + silica + PDMS grafting	/	ΔT = 53 °C (1 sun)	Prevents icing at −50 °C; Melts frost/ice: 300 s	Self-healing after plasma; Long-term stability; Eco-friendly; Scalable; Self-cleaning	[132]
CB-PDMS microarray	Photolithography + CB infusion	151.1 ± 0.9°	Freezing: delay 87%; ΔT not specified	Deicing time reduced by ~43.1% vs. control	Flexible, stable under bending, and friction; Photolithographic precision; Cost-effective	[133]
MP@SHS (PDMS + CNPs with micropores)	Salt-template casting	153.5 ± 0.5°	T ↑ to 75 °C (360 s, 1 sun); Absorbance = 98.3%	Freezing delay: ~648 s; Melting: ~129 s	Resistant to chemical/mechanical damage; Flexible	[134]

**Table 4:** Research on polymer-based photothermal superhydrophobic surfaces.

Materials	Preparation Method	Water Contact Angle	Photothermal Performance	Anti-Icing/Deicing Ability	Durability& Other Features	Ref.
PPy-coated basalt (BP), SiO_2_ NPs, fluorocarbon resin	Two-step spray with inverse infusion process	155° ± 2°	Ice removal in 309 s (frost)/760 s (ice cube) under 1 sun	Icing delay: 656 s; Ice adhesion < 20 kPa after cycles	Stable after 300 sandpaper + 100 tape cycles	[135]
PET fabric, PPy, pentaerythritol tetraacrylate, octadecyl acrylate, APTES	In situ oxidative polymerization + 1,4-conjugation addition	155.8°	T ↑ to 91 °C (simulated sunlight, 20 mA)	Not directly measured; Fabric suitable for photothermal deicing	Stable after UV, acid/base, organics, 25 abrasion cycles, 8 wash cycles; Self-cleaning; Fluorine-free	[136]
PPy/ATP@hexadecylPOS, silicone resin	Spray-coating PPy/ATP+silicone resin on Al	162.7°	ΔT = 70 °C (3 min, 1 sun)	Fast deicing; Long-term anti-icing (4 weeks)	Mechanical, chemical, and thermal stability; Outdoor-tested; Fluorine-free; Eco-friendly; Hierarchical porous structure	[137]
F-SiO_2_@Tp/Fe, silicone resin	One-step spray-coating	159°	ΔT = 81 °C (1 sun)	Icing delay ×3, stable de/defrosting cycles	Stable under abrasion, peeling, and corrosion	[138]
PU sponge, Fe_3_O_4_ NPs, PDA, fluorosilane	Deposition of Fe_3_O_4_ + PDA + fluorination	≈150°	Surface T reached ~51 °C (1 sun)	No icing under 0.3 sun at −30 °C; Melts ice in 10 min under 1 sun	Self-cleaning; Self-healing; Stable in harsh conditions; Porous structure	[139]
Cotton fabric, PDA, PEI, GA@AgNPs, PDMS	Dipping and sequential coating	159.6°	Surface T reached 70.4 °C (simulated sunlight, 20 A)	/	Resistant to washing, abrasion, chemicals, and high temperatures; Environmentally friendly; Low-cost	[140]
Dual-size LMNSs, epoxy resin, F13–TMS	Gravity-assisted sedimentation + immersion coating	166.1° ± 0.8°,	T ↑ from ~13 °C to 112 °C in 60 s (laser)	Not specifically measured but implied to be effective	Abrasion distance > 320 cm, 210 tape cycles; Chemical/thermal Resistance; Renewable	[141]
PAN (AgNP-loaded), PVDF-PDMS	Electrospinning + immersion + reduction	/	Energy efficiency 75.2%; T sufficient for membrane distillation	Not tested for icing, tested for desalination	Stable over 10 h of distillation with low conductivity	[143]

## Data Availability

No primary research results, software, or code have been included, and no new data were generated or analyzed as part of this review.
